# Multiplexed single-cell analysis reveals prognostic and nonprognostic T cell types in human colorectal cancer

**DOI:** 10.1172/jci.insight.154646

**Published:** 2022-04-08

**Authors:** Kazuya Masuda, Adam Kornberg, Jonathan Miller, Sijie Lin, Nathan Suek, Theo Botella, Kerim A. Secener, Alyssa M. Bacarella, Liang Cheng, Matthew Ingham, Vilma Rosario, Ahmed M. Al-Mazrou, Steven A. Lee-Kong, Ravi P. Kiran, Marlon Stoeckius, Peter Smibert, Armando Del Portillo, Paul E. Oberstein, Peter A. Sims, Kelley S. Yan, Arnold Han

**Affiliations:** 1 Columbia Center for Translational Immunology,; 2 Department of Microbiology & Immunology,; 3 Department of Pediatrics,; 4 Columbia Center for Human Development,; 5 Department of Medicine, Division of Hematology & Oncology,; 6 Herbert Irving Comprehensive Cancer Center, and; 7 Department of Surgery, Division of Colorectal Surgery, Columbia University, New York, New York, USA.; 8 New York Genome Center, New York, New York, USA.; 9 Department of Pathology,; 10 Departments of Systems Biology and Biochemistry & Molecular Biophysics,; 11 Department of Medicine, Division of Digestive & Liver Diseases, and; 12 Department of Genetics & Development, Columbia University, New York, New York, USA.

**Keywords:** Immunology, Cancer immunotherapy, Colorectal cancer, T cells

## Abstract

Clinical outcomes in colorectal cancer (CRC) correlate with T cell infiltrates, but the specific contributions of heterogenous T cell types remain unclear. To investigate the diverse function of T cells in CRC, we profiled 37,931 T cells from tumors and adjacent normal colon of 16 patients with CRC with respect to transcriptome, TCR sequence, and cell surface markers. Our analysis identified phenotypically and functionally distinguishable effector T cell types. We employed single-cell gene signatures from these T cell subsets to query the TCGA database to assess their prognostic significance. We found 2 distinct cytotoxic T cell types. *GZMK*^+^KLRG1^+^ cytotoxic T cells were enriched in CRC patients with good outcomes. *GNLY*^+^CD103^+^ cytotoxic T cells with a dysfunctional phenotype were not associated with good outcomes, despite coexpression of CD39 and CD103, markers that denote tumor reactivity. We found 2 distinct Treg subtypes associated with opposite outcomes. While total Tregs were associated with good outcomes, CD38^+^ Tregs were associated with bad outcomes independently of stage and possessed a highly suppressive phenotype, suggesting that they inhibit antitumor immunity in CRC. These findings highlight the potential utility of these subpopulations in predicting outcomes and support the potential for novel therapies directed at CD38^+^ Tregs or CD8^+^CD103^+^ T cells.

## Graphical abstract

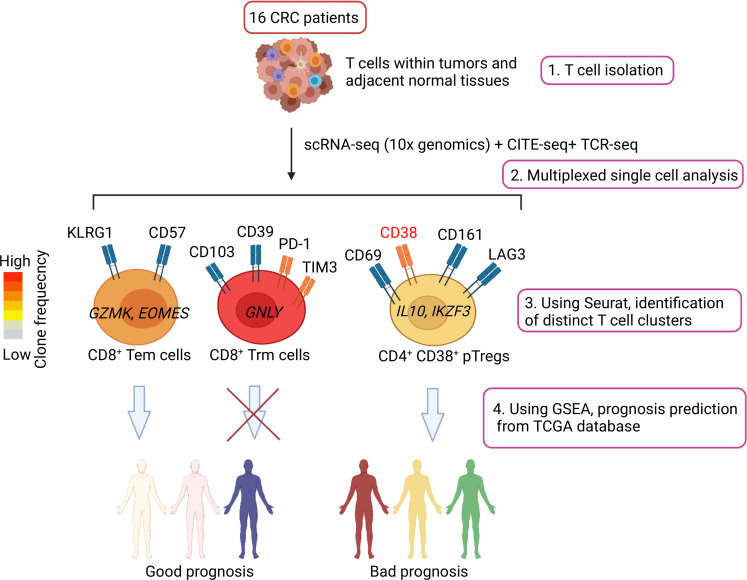

## Introduction

Colorectal cancer (CRC) is a highly prevalent cancer and a leading cause of cancer deaths worldwide (1). Currently, clinical outcomes in CRC are broken down by staging criteria that guide treatment and prognosis. However these are imperfect criteria due to the large variability in survival outcomes, especially in Stage II and Stage III patients (2). Though landmark histological studies correlated T cell infiltration with effector memory CD45RO^+^CD8^+^ T cells with survival in CRC (3, 4), the success of T cell targeting immunotherapy in CRC has been limited to a rare subset with high microsatellite instability (microsatellite instability–high [MSI-H] tumors that only represent ~15% of CRC) that are characterized by a higher degree of mutation due to defective DNA mismatch repair (5–9). Thus, an improved molecular understanding of T cell infiltrates within cancers may enhance our ability to develop novel T cell–directed immunotherapies for treatment of CRC.

Single-cell transcriptome analyses of T cell infiltrates have been performed in several types of tumors, including breast cancer (BRC), liver cancer, and CRC (7, 10–13). However, the phenotype and function of T cell subtypes in CRC as they relate to patient prognosis remain unclear. While CD8^+^ T cell infiltrates have been shown to predict good outcomes, the role of Tregs is controversial (14, 15). In many tumor types, including breast, lung, gastric, and hepatocellular carcinomas, Treg infiltration correlates with poor outcomes (16–20). In CRC, there is a lack of consensus, with multiple studies concluding that Treg density correlates with good outcomes in CRC (21, 22), while others have reported that Tregs promote tumor progression by suppressing antitumor immunity (23). Such conflicting data may be due, at least in part, to heterogeneity within Tregs in CRC (24).

To probe the functional and phenotypic heterogeneity of T cell infiltrates and their contribution to clinical outcomes in CRC, we performed unbiased droplet-based single-cell RNA-Seq (scRNA-Seq) of T cells isolated from tumors or adjacent normal colon of 16 treatment-naive CRC patients (25). We used a modified workflow for scRNA-Seq that incorporated oligonucleotide-tagged antibodies to assess cell surface protein expression levels in T cells, termed CITE-Seq (Cellular Indexing of Transcriptomes and Epitopes by Sequencing), and also to distinguish cells from different samples to enable multiplexing, termed Cell Hashing (26, 27). To assess the functional capacity of T cells, including cytokine production, a portion of cells from each sample was stimulated with PMA/ionomycin prior to analysis. To examine the prognostic significance of diverse T cell populations, we used semiquantitative gene set enrichment analysis (GSEA), which enabled us to assess the enrichment of single-cell gene signatures within bulk RNA expression profiles published in The Cancer Genome Atlas (TCGA) cohorts of several types of tumors, including CRC, BRC, and melanoma (28–31). Using this comprehensive approach, we have defined with enhanced granularity T cell subpopulations responsible for the observed clinical outcomes in CRC, which also highlights their potential for predicting patient survival independent of cancer staging. We also identified potentially novel candidate T cell subpopulations that may be therapeutically targeted to improve upon existing immunotherapies for CRC.

## Results

### Single-cell profiling of T cells in CRC and adjacent normal colon.

**Figure 1:**
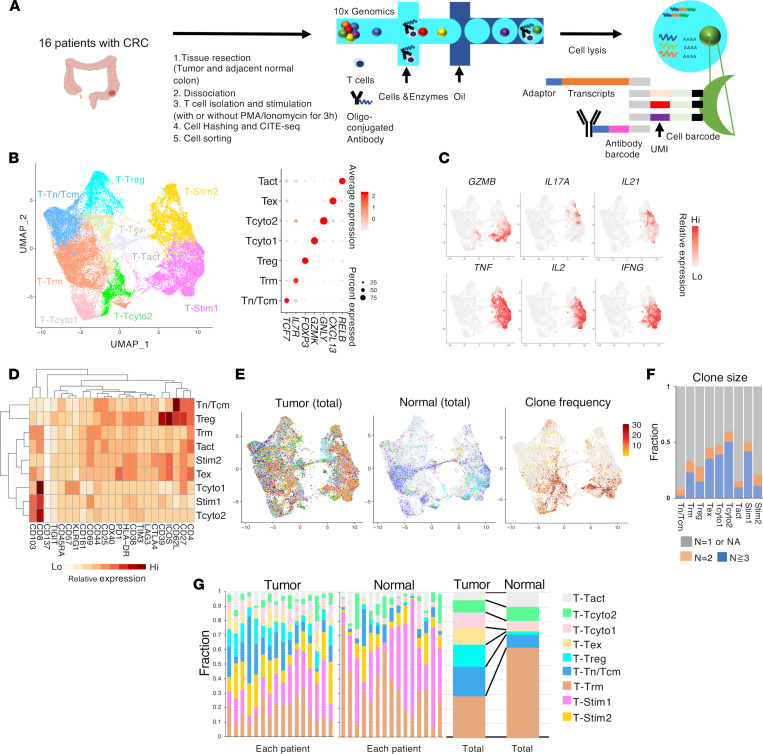
Characterization of T cells in CRC and normal adjacent colon by multiplexed scRNA-Seq. (**A**) Workflow for preparation of multiplexed scRNA-Seq and TCR-Seq libraries using the 10× Genomics platform. (**B**) Uniform Manifold Approximation and Projection (UMAP) of 35,145 single T cells in CRC and adjacent normal colon from a total of 16 patients (left). Unsupervised clustering of single-cell transcriptomes was colored by each cluster. Dot plot depicting relative expression of identifying marker genes for clusters (right). (**C**) Heatmap of cytokines and effectors based on relative gene expression. (**D**) Heatmap depicting average ADT signal in the CITE-Seq antibody panel within each cluster. (**E**) UMAP showing distribution of single T cells within tumor (left) or adjacent normal colon (middle) colored by individual patients. Color and number of tumor-infiltrating T cells or normal cells from each patient are indicated in Supplemental Table 4. Cells are colored based on TCR clone frequency (right), normalized to total number of T cells per patient sample. (**F**) Bar graph depicting clonal composition of T cells within each cluster. (**G**) Bar graphs showing relative distribution of cells from each cluster (as indicated) within tumor (left) or normal adjacent colon (middle) of each patient, and proportion of cells within each cluster from total tumor or normal tissues (right).

We obtained matched CRC and adjacent normal colon tissue specimens from patient surgical resections for comprehensive single-cell analysis. Our methodology offered the major advantage that it enables multiplexing to minimize experimental variability/batch effects. Here, we sorted and analyzed 37,931 viable TCRαβ^+^CD3^+^ cells from CRC and adjacent normal colon from 16 patients with respect to single-cell transcriptome, 23 cell surface marker protein levels (CITE-Seq), and T cell antigen receptor (TCR) sequence clonality (Figure 1A and Supplemental Figure 1A; supplemental material available online with this article; https://doi.org/10.1172/jci.insight.154646DS1) (26). A portion of each sample was also subjected to 3-hour stimulation with PMA/ionomycin. Cells from individual normal colon versus tumor tissue specimens or stimulated versus nonstimulated fractions were then resolved through labeling with distinct antibody hashtags (27). After stringent quality filtering, we obtained 35,145 single T cell transcriptomes. TCR-Seq analysis assigned annotated TCR sequences for 32,550 single T cells, of which 27,999 cells had paired TCRαβ sequences (see Methods).

Unsupervised clustering of the single-cell transcriptomes generated 9 distinct T cell clusters, of which 7 clusters were populated by nonstimulated T cells versus 2 clusters populated by stimulated T cells (Figure 1B and Supplemental Figure 1B). Those clusters populated by unstimulated T cells were identified by their canonical marker expression (Figure 1B). Stimulated T cells formed 2 clusters, T-Stim1 and T-Stim2, composed primarily of CD8^+^ and CD4^+^ T cells, respectively (Supplemental Figure 1, B and C). These 2 clusters were distinguishable by the effector molecules and cytokines they produced, including *GZMB*, *IL2*, *TNFA*, *IFNG*, *IL21*, and *IL17A* (Figure 1C). While stimulation did induce common global changes in effector gene expression, which led stimulated cells to cluster separately, the fundamental character and phenotype of identified T cell types did not change (32). Therefore, distinct unstimulated T cell phenotypes could be matched to cognate-stimulated T cell phenotypes through common gene expression, adding significant functional information to our phenotypic data.

The total naive/central memory (T_Tn/Tcm) cluster was enriched in markers of more naive T cell phenotypes (*TCF7*, *CCR7*, CD62L^+^, CD45RA^+^, CD27^+^) (Figure 1D and Supplemental Figure 1, D and E). There was scant clonal expansion observed within this population (Figure 1, E and F). The T_Treg cluster consisted primarily of CD4^+^ T cells and was enriched in markers associated with Tregs (*FOXP3*, *BATF*, CD25^+^, CD62L^+^, CTLA4^+^, CD27^+^, ICOS^+^, TIGIT^+^, OX40^+^, TIM3^+^) (Figure 1D and Supplemental Figure 1, C–E). Both T_Trm (resident memory T) and T_Tact (activated T) clusters were enriched in markers of Trm cells (CD69^+^, CD103^+^) (Figure 1D and Supplemental Figure 1E). The T_Trm cluster, which was the most diverse in terms of gene expression, consisted of both CD4^+^ and CD8^+^ T cells with distinct signature genes (*IL7R*, *JAML*, *ITGA1*) and was widely present in both normal colon and tumors (Figure 1, B and E, and Supplemental Figure 1, C and D). The T_Tact cluster exhibited signatures of T cell activation (*RELB*, *NFKB2*, CD25^+^, CD69^+^, CD27^+^) (Figure 1D and Supplemental Figure 1, D and E). The T_Tex (exhausted T) cluster was enriched for expression of genes (*CXCL13*, *HAVCR2*) and surface proteins (PD-1, ICOS) associated with T cell exhaustion or dysfunction (33) (Figure 1D and Supplemental Figure 1, D and E). Notably, cells within this cluster were highly clonally expanded and coexpressed CD39 and CD103, consistent with a T cell phenotype with tumor reactivity (34, 35) (Figure 1D and Supplemental Figure 1E).

We identified 2 clusters of cytotoxic T cells, T_Tcyto1 (cytotoxic T1) and T_Tcyto2 (cytotoxic T2), expressing signature genes of effector function and cytotoxicity (Figure 1B and Supplemental Figure 1D). The T_Tcyto1 cluster was enriched for markers of effector memory T cells (Tem; *EOMES*, *GZMK*, KLRG1^hi^, CD57^hi^, CD27^hi^, CD44^+^), while the T_Tcyto2 cluster (*GNLY*, *GZMH*, *PRF1*) was enriched for expression of the resident memory marker CD103 (Figure 1D and Supplemental Figure 1, D and E). Both clusters consisted predominantly of CD8^+^ T cells with high clonal expansion, although CD4^+^ T cells were present in both populations (Figure 1D and Supplemental Figure 1C). The T_Tcyto1 cluster, but not the T_Tcyto2 cluster, was relatively enriched within tumors compared with normal colon (Figure 1G and Supplemental Figure 2A). The presence of these distinct cytotoxic CD8^+^ T cell subsets, distinguished by expression of CD57 or CD103, was validated by IHC (Supplemental Figure 2B).

Strikingly, T cells from normal colon exhibited relatively less phenotypic diversity and were essentially absent in T_Treg and T_Tex clusters (Figure 1G and Supplemental Figure 2A). In contrast, intratumoral T cells were widely distributed across all clusters, and each T cell subtype was present in each individual patient tumor (Figure 1G and Supplemental Figure 2C), suggesting that functional insights and gene signatures gleaned through this analysis are broadly applicable in human CRC.

### Prognostic significance of distinct T cell subsets with effector functions in CRC.

**Figure 2:**
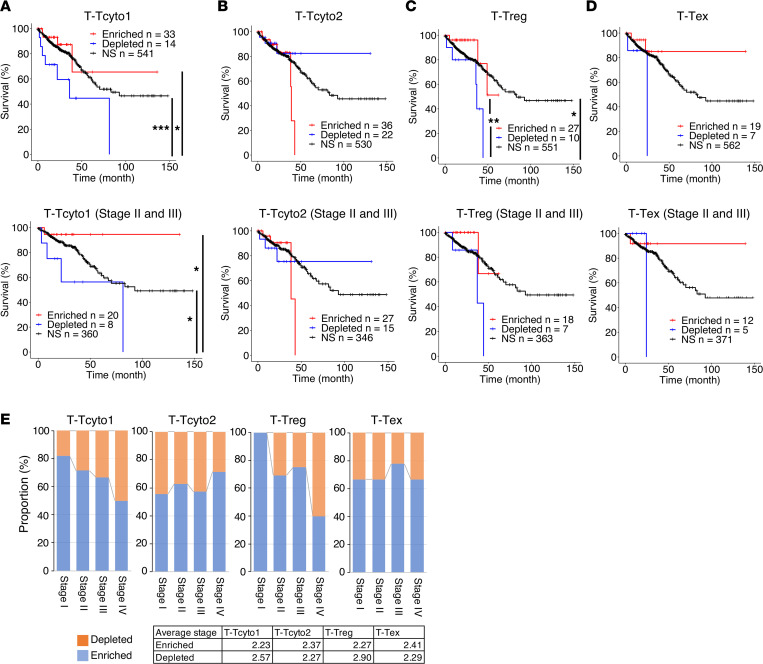
Clinical outcomes associated with single T cell subtypes in CRC. (**A**–**D**) Kaplan-Meier curves of overall survival in the CRC TCGA cohort for patients enriched or depleted for the following gene sets T_Tcyto1 (**A**), T_Tcyto2 (**B**), T_Treg (**C**), T_Tex (**D**). Kaplan-Meier curves from enriched or depleted patients with all stages (upper) or only those patients with Stage II and III tumors (lower). *P < 0.05; **P < 0.02; ***P < 0.001 (log-rank test). See detailed *P* values in Supplemental Table 6. (**E**) Bar graph depicting relative proportion of patients by stage that are enriched or depleted for T_Tcyto1, T_Tcyto2, or T_Treg gene sets. Average stage of patients is indicated in table.

To investigate the prognostic significance of distinct T cell subtypes, we applied semiquantitative GSEA to the TCGA database (29). We queried the top 30–40 differentially expressed genes in nonstimulated clusters with effector function (T_Tcyto1, T_Tcyto2, T_Tex, and T_Treg) within published bulk RNA-Seq data from the CRC TCGA cohort (Supplemental Table 5). We subsequently compared survival between the statistically gene set–enriched and –depleted patients through Kaplan-Meier analysis (Figure 2, A–D). Importantly, CRC patients (all stages) with enrichment of the T_Tcyto1 gene set (*n* = 33) exhibited a good prognosis compared with those with depletion (*n* = 14, *P* < 0.05) (Figure 2A). Although stage influenced survival as expected (Supplemental Figure 3A), our disaggregated data demonstrated that patients with Stage II and Stage III tumors who also had T_Tcyto1 enrichment exhibited improved survival compared with those with depletion (Figure 2A). Thus, our data suggest that enrichment of T_Tcyto1 gene signatures within tumors correlates with improved survival independent of stage. However, there was no statistically significant difference in survival between T_Tcyto2 gene set–enriched (*n* = 36) and –depleted patients (*n* = 22, *P* = 0.2) (Figure 2B). T_Tcyto2 enrichment or depletion did not correlate with differences in survival in those patients with Stage II and Stage III tumors (Figure 2B), in contrast to T_Tcyto1. These results suggest that T_Tcyto1 and T_Tcyto2 clusters contain T cells with functionally distinct roles in CRC.

Since a cytotoxic T cell type with a similar phenotype has been reported to correlate with a positive outcome in melanoma (36), we queried gene sets from the T_Tcyto1 and T_Tcyto2 clusters in gene expression data from the TCGA cohort of melanoma (Supplemental Table 5 and Methods). In the melanoma cohort, positive clinical outcomes were associated with both T_Tcyto1 (*P* < 0.05) and T_Tcyto2 (*P* < 0.05) gene sets, and there was higher overlap between gene set–enriched patients (86 %, T_Tcyto1; 78 %, T_Tcyto2) than the CRC cohort, likely reflecting differences in cytotoxic T cell populations between these 2 cancers (Supplemental Figure 3, B–D). Accordingly, resident memory phenotype CD8^+^ T cells have been correlated with positive prognosis in melanoma but not CRC (37). Enrichment of T_Tcyto1 or T_Tcyto2 gene signatures in melanoma patients was not likely to be dependent on stage (Supplemental Figure 3, A and E).

T_Treg cluster gene set–enriched patients showed a significantly better prognosis than the –depleted patients (*P* < 0.05) in CRC, although this cluster did not show any prognostic significance in melanoma (Figure 2C and Supplemental Figure 3F). These findings are consistent with the preponderance of histologic studies that concluded that Tregs are associated with favorable outcomes in CRC (21, 22, 38, 39). Enrichment of T_Treg genes was relatively higher in patients with early-stage cancer, indicating that the improved prognosis may be related to stage (Figure 2, C and E). Analysis of the prognostic value of the T_Tex gene set did not reach statistical significance in CRC (Figure 2D).

### Distinct prognostic effector memory and nonprognostic resident memory CD8^+^ T cell infiltrates.

**Figure 3:**
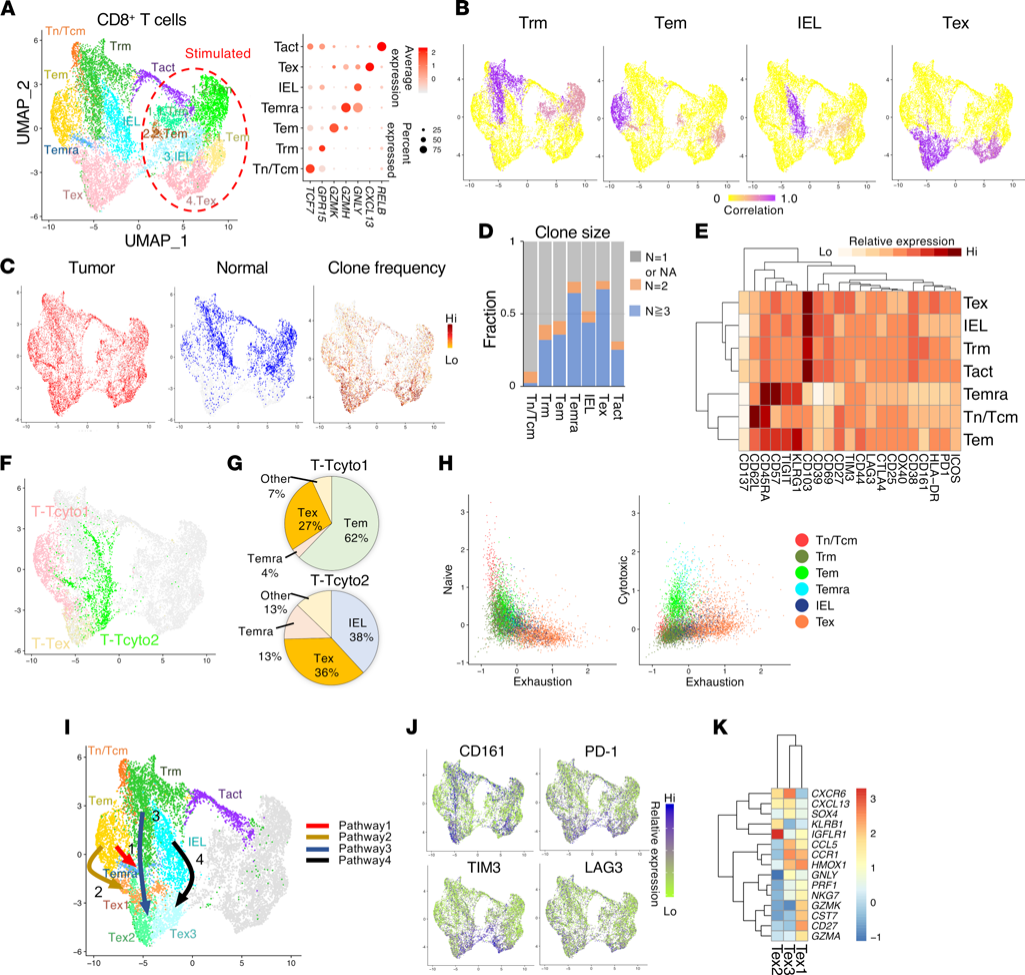
Distinct phenotypes of prognostic or nonprognostic CD8 T infiltrates in CRC. (**A**) UMAP of 19,183 single CD8^+^ T cells in CRC and adjacent normal colon from a total of 16 patients with unsupervised clustering. Colored by cluster. Distinct clusters contain unstimulated T cells and stimulated T cells (left). Dot plot depicting relative expression of identifying marker genes for each nonstimulated cluster (right). (**B**) Pearson correlation map between each cluster in nonstimulated and stimulated cells, correlated based on 21 ADT signals. (**C**) Cells from tumor (red) or adjacent normal colon (blue) are indicated in the UMAP in **A** (left). Cells are colored based on TCR clone frequency (right), normalized to total number of T cells per patient sample. (**D**) Bar graph depicting clonal composition of T cells within each cluster. (**E**) Heatmap depicting average ADT signal in the CITE-Seq antibody panel within each nonstimulated cluster. (**F**) Distribution of cells within the clusters T_Tcyto1, T_Tcyto2, and T_Tex (in Figure 1B) plotted on the CD8 UMAP. (**G**) Pie charts depicting composition of cells within the clusters T_Tcyto1 or T_Tcyto2 as a proportion of CD8 clusters CD8_Tem, CD8_Temra, CD8_IEL, and CD8_Tex. (**H**) Dot plots of cells from each CD8 nonstimulated cluster by naive versus exhaustion score or cytotoxic versus exhaustion score. (**I**) Distinct differentiation pathways of CD8^+^ T cells based on cell trajectory (relate to Supplemental Figure 4, F and G). (**J**) Heatmap of ADT signals (as indicated) on the CD8 UMAP. (**K**) Gene heatmap of differentially expressed genes within Tex subpopulations (Tex1–Tex3) in **I**.

To define T cells with improved granularity, we segregated CD8^+^ and CD4^+^ T cells based on antibody-derived tag (ADT) signal and transcriptome, and we analyzed them separately (Supplemental Figure 4A). Within 12,642 single CD8^+^ T cells, unsupervised clustering based on transcriptome identified 13 distinct clusters consisting of 7 distinct nonsimulated populations and stimulated CD8^+^ T cells (Figure 3A). Those clusters populated by unstimulated T cells were identified by their canonical marker expression (Figure 3A). Based on 21 ADT signals, we were able to link nonstimulated clusters to cognate-stimulated clusters (Figure 3B). Overall, all CD8 subtypes were observed within tumors, while cells from normal tissue were largely confined to clusters CD8_Trm, CD8_IEL, and CD8_Tact (Figure 3C).

Within nonstimulated cells, the CD8_Tn/Tcm cluster contained cells with relatively higher *TCF7* expression and the least clonal expansion (Figure 3D and Supplemental Figure 4, B and C). Predominantly CD8^+^ Trm cells (CD69^+^, CD103^+^) comprised the CD8_Trm, CD8_intraepithelial lymphocyte (IEL), and CD8_Tact clusters (Figure 3E). The CD8_Trm cluster (*GPR15*, *IL7R*) mainly contained cells with relatively high expression of CD161, described to define a particular memory cell type in CD8^+^ T cells, including mucosal-associated invariant T (MAIT) cells expressing the semiinvariant TCR Vα7.2-Jα33/12/20 (40); these cells were present at low frequency (~4 %) within this cluster (Figure 3E, Supplemental Figure 4B, and Supplemental Table 7). Cells within cognate-stimulated clusters (CD8_Stim1.1.Trm, CD8_Stim1.2.Trm) expressed cytokines including *TNF*, *IL2*, and *IFNG* (Figure 3A and Supplemental Figure 4D).

Cells within the CD8_IEL cluster expressed effectors (*GNLY*, *PRF1*) with expression of IEL signature genes including killer cell immunoglobulin-like receptors (KIRs; *KIR3DL1/2*) (Figure 3A and Supplemental Figure 4B) (41). Its cognate-stimulated cluster (CD8_Stim3.IEL) contained cells expressing effector molecules *GNLY*, *GZMB*, and *PRF1* (Figure 3A and Supplemental Figure 4D). The CD8_IEL cluster overlapped with the total T cell T_Tcyto2 cluster, which accounted for 38% of cells within the cluster (Figure 3, F and G). The CD8_Tact cluster consisted of activated T cells (*NFKB2*, *RELB*) and contained both stimulated and nonstimulated cells (Figure 3A and Supplemental Figure 4B). The CD8_IEL and CD8_Tact clusters had relatively high expression of CD39 and CD103 (Supplemental Figure 4E).

The CD8_Tem cluster contained Tem cells (*EOMES*, *GZMK*, *CXCR3*, KLRG1^+^, CD57^+^, CD44^+^, CD27^+^) with relatively higher expression of naive T cell markers (*CCR7*, *TCF7*), similar to “effector” T cells previously described in several types of tumors (Figure 3, E and H; Supplemental Figure 4B; and Supplemental Table 8) (11, 33, 42, 43). Cells within its cognate-stimulated cluster, CD8_Stim.Tem, expressed *IL2*, *TNFA*, and *IFNG* (Figure 3A and Supplemental Figure 4D). The CD8_Tem cluster largely overlapped with the positively prognostic total T cell T_Tcyto1 cluster, which constituted 62% of the cells within this cluster (Figure 3, F and G).

The CD8_Temra cluster contained terminally differentiated effector memory CD45RA^+^ (Temra) cells (*GZMH*, *GNLY*, CD45RA^hi^, KLRG1^hi^, CD57^hi^, TIGIT^hi^, CD27^–^) with high clonal expansion and enriched for “cytotoxic” signature genes (*FGFBP2*, *GZMH*) (Figure 3, D, E, and H; Supplemental Figure 4B; and Supplemental Table 8) (42). The CD8_Tex cluster was enriched for Tex cell markers (*HAVCR2*, *CXCL13*, PD-1^+^, TIM3^+^) and contained highly clonally expanded cells coexpressing CD39 and CD103 (Figure 3, D, E, and H; Supplemental Figure 4, B and E; and Supplemental Table 8). Cells within its cognate-stimulated CD8_Stim4.Tex cluster were capable of producing certain effectors (*PRF1*, *GZMB*, *IFNG)* with relatively low *IL2* and *TNFA* expression (Supplemental Figure 4D). Notably, the nonprognostic T_Tcyto2 cluster contained a large number of Tex cells (36%) as well as IEL-like cells (38%), suggesting that these cells are dysfunctional within tumors (Figure 3, F and G).

To visualize the relationship between CD8^+^ T cell clusters, we used diffusion maps and ordered cells in pseudotime within nonstimulated clusters (Supplemental Figure 4F). In combination with TCR sharing data, we found 4 differentiation pathways of CD8^+^ T cells within tumors, which culminated in terminally differentiated distinct cell types present within CD8_Temra or CD8_Tex clusters (Figure 3, I–K, and Supplemental Figure 4, F and G). In one pathway (pathway 1), CD8_Tem cells differentiated into CD8_Temra cells. The other pathways culminated into 3 distinct Tex subpopulations with coexpression of CD39 and CD103, suggestive of tumor reactivity: pathway 2, CD8_Tem cells toward CD8_Tex1 subpopulation (*NKG7*, *GZMK*, *GZMA*); pathway 3, CD8_Trm cells toward CD8_Tex2 subpopulation (*KLRB1*, *IGFLR1*, CD161^hi^); or pathway 4, IELs toward CD8_Tex3 subpopulation (*CXCR6*, *GNLY*, *CCL5*, CD161^+^). Interestingly, cells within the positively prognostic T_Tcyto1 cluster were largely present in the CD8_Tem and Tex1 clusters with more effector function, while cells within the nonprognostic T_Tcyto2 cluster were present in the CD8_IEL and Tex3 clusters containing more dysfunctional cells (Figure 3, F, G, and I). Thus, diverse types of effector CD8^+^ T cells (prognostic Tem and nonprognostic Trm cells) transitioned to terminally differentiated cells through different pathways within tumors.

### Phenotypically and functionally diverse CD4^+^ T cell infiltrates.

**Figure 4:**
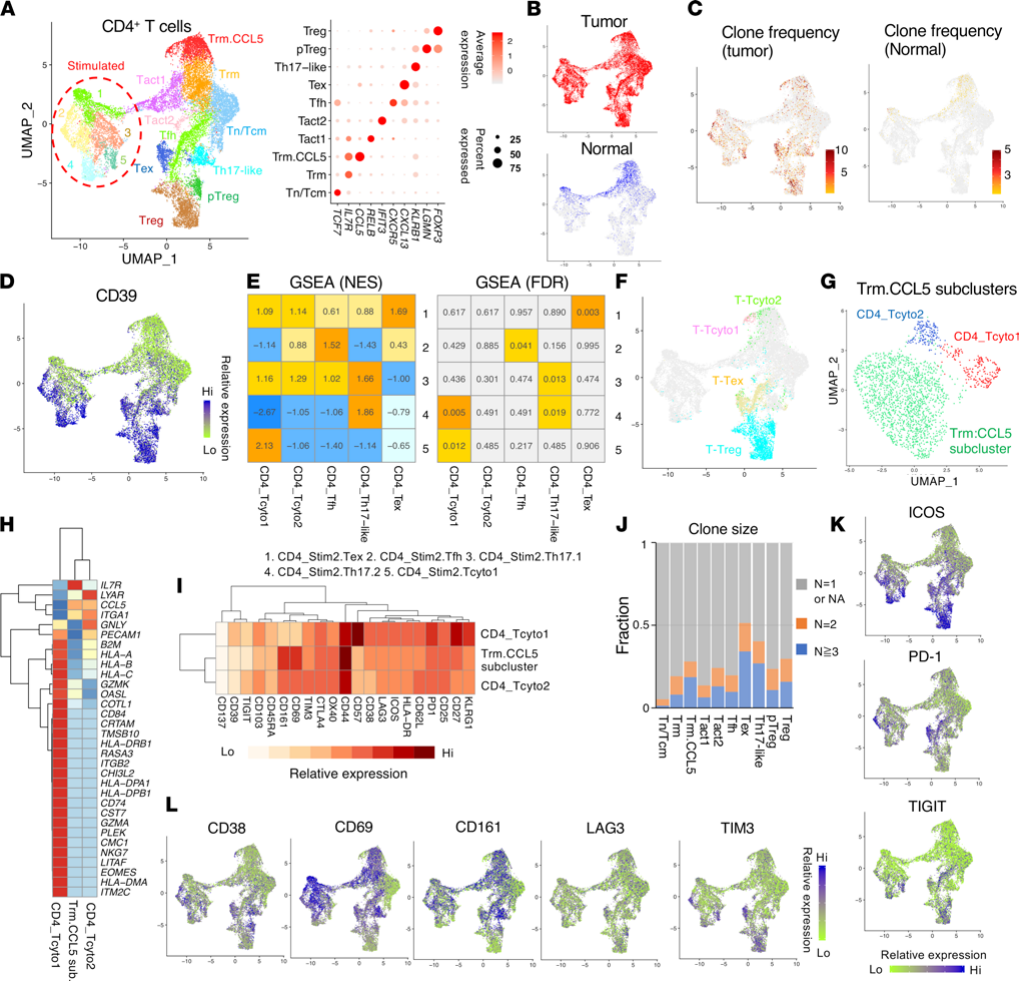
The functional and phenotypic diversity of single CD4^+^ T cells with prognostic significance in CRC. (**A**) UMAP of 12,642 single CD4^+^ T cells in CRC and adjacent normal colon from a total of 16 patients (left). Colored by each cluster. Distinct clusters contain unstimulated T cells and stimulated T cells. Dot plot depicting relative expression of identifying marker genes for each nonstimulated cluster (right). (**B**) Cells from tumor or adjacent normal colon are indicated on the CD4 UMAP. (**C**) Heatmap of TCR clone frequency in intratumoral T cells or T cells from normal tissue on the CD4 UMAP. (**D**) Heatmap of CD39 ADT signal on the CD4 UMAP. (**E**) GSEA based on gene expression between nonstimulated clusters and stimulated subclusters (as indicated). (**F**) Distribution of cells within the clusters of T_Tcyto1, T_Tcyto2, T_Tex, and Treg (identified in Figure 1B) on the CD4 UMAP. (**G**) Subclustering of CD4_Trm.CCL5 cluster. Three subpopulations are labeled. (**H**) Heatmap showing relative gene expression of the genes indicated within each CD4_Trm.CCL5 subcluster shown in **G**. (**I**) Heatmap depicting average ADT signal in the CITE-Seq antibody panel within each CD4_Trm.CCL5 subcluster shown in **G**. (**J**) Bar graph depicting clonal composition of T cells within each cluster. (**K** and **L**) Heatmap of ADT signals (as indicated) on the CD4 UMAP.

Unsupervised clustering of 19,183 single CD4^+^ T cells based on transcriptome identified 15 clusters consisting of 10 distinct nonstimulated populations and stimulated T cells (Figure 4A and Supplemental Figure 5A). Those clusters populated by unstimulated T cells were identified by their canonical marker expression (Figure 4A). Although all clusters contain intratumoral CD4^+^ T cells, normal CD4^+^ T cells were confined to nonstimulated clusters (CD4_Trm, CD4_Trm.CCL5, CD4_Tact1) and stimulated clusters (CD4_Stim1, CD4_Stim3) with a resident memory phenotype (*IL7R*, CD69^+^, CD161^+^) (Figure 4B and Supplemental Figure 5, A and B). Compared with intratumoral CD4^+^ T cells, there was limited clonal expansion in normal colon CD4^+^ T cells (Figure 4C). Stimulated normal CD4^+^ T cells expressed *IL2*, *IFNG*, *TNFA*, and *IL10* in addition to the Th1 marker *Tbx21* (CD4_Stim1) or Tfh markers *BCL6* and *IL21* (CD4_Stim3) (Supplemental Figure 5, C and D). Gene markers associated with Th17 cells or Tregs (*FOXP3*, *RORC*, *IL17*) were mostly absent in normal colon T cells (Supplemental Figure 5C).

Intratumoral CD4^+^ T cells primarily consisted of effector T cells expressing the activation marker CD39 (Figure 4D). Many canonical genes that define effector CD4^+^ Th subsets were elicited through stimulation, including Th1, Th17, and Tfh signature genes (Supplemental Figure 5C). The CD4_Stim2 cluster (including distinct T subsets) was further subdivided into 5 distinct subclusters by unsupervised clustering (Supplemental Figure 5, E and F). Through GSEA, we were able to link these subclusters with their cognate nonstimulated clusters and named them accordingly (Figure 4E).

Interestingly, CD4^+^ T cells within cytotoxic T_Tcyto1 and T_Tcyto2 clusters were observed in the CD4_Trm.CCL5 cluster expressing *CCL5*, known to regulate proliferation and homeostatic maintenance of tissue Trm cells (44) (Figure 4F). Upon further subclustering of CD4_Trm.CCL5, CD4_Tcyto1 and CD4_Tcyto2 subclusters predominantly contained T_Tcyto1 cells and T_Tcyto2 cells, respectively (Figure 4G). Cells within the CD4_Tcyto1 subcluster (*NKG7*, *EOMES*, *GZMK*, *GZMA*, KLRG1^+^, CD57^+^, CD27^+^, PD-1^+^) were enriched within tumors and exhibited a similar phenotype to the CD8_Tem cluster (Figure 4, H and I, and Supplemental Figure 5G). Its cognate-stimulated cluster (CD4_Stim2.Tcyto1) contained cells expressing effector cytokines *IFN* and *IL2* and chemokines (*CCL3*, *CCL4*) that can mediate T cell recruitment (45) (Supplemental Figure 5F). Likewise, cells within CD4_Tcyto2 exhibited a similar phenotype to the T_Tcyto2 cluster, and were not confined to tumors (Figure 4, H and I, and Supplemental Figure 5G).

The CD4_Th17-like cluster contained cells with Th17 cell signature genes (*KLRB1*, *RORA*, *CXCR6*) with high clonal expansion, and its cognate-stimulated subclusters, (CD4_Stim2.Th17.1 and CD4_Stim2.Th17.2) contained cells expressing canonical Th17 genes (*RORC*, *IL17A*) (Figure 4J and Supplemental Figure 5, B, C, E, and F). Cells within the CD4_Stim2.Th17.1 cluster expressed genes associated with regulatory function (*IL10*, *ITGA2*) (46, 47), whereas cells within the CD4_Stim2.Th17.2 cluster expressed proinflammatory effectors (*IFNG*, *IL23R*, *PRF1*), described to be produced by pathogenic Th17 cells in autoimmunity (Supplemental Figure 5F) (48).

The positively prognostic *FOXP3*^+^ T cell population (T_Treg) was largely divided into 2 CD4 subpopulations (CD4_Treg, CD4_pTreg) (Figure 4A). The CD4_Treg cluster contained cells expressing distinct signature genes (*BATF*, *GATA3*, *IKZF2*) and high levels of ICOS (Figure 4K and Supplemental Figure 5B). The transcription factor Helios (encoded by the gene *IKZF2*) has been proposed to mark thymically derived Tregs, though this is currently controversial (49–51). In contrast, Helios^–^ Tregs within the CD4_pTreg (peripherally derived) cluster exhibited a distinct phenotype (CD161^+^, CD69^+^, LAG3^+^, CD38^hi^) and shared genes (*IL26*, *KLRB1*, *IKZF3*) with peripherally derived CD4^+^*FOXP3*^–^ T conventional (Tconv) cells (Figure 4L and Supplemental Figure 5H) (52–55).

The CD4_Tfh cluster contained PD-1^hi^ICOS^hi^CD27^hi^*CXCL13*^+^ T cells expressing naive T cell markers (*CCR7*, *TCF7*) and correlated with its stimulated clusters (CD4_Stim2.Tfh) expressing Tfh signature genes (*BCL6*, *CXCR5*, *IL21*) (Figure 4K and Supplemental Figure 5, B–D and F). The CD4_Tex cluster (*CXCL13*, *GNLY*, *GZMA*, TIGIT^hi^, ICOS^hi^, CD27^–^), which expressed coinhibitory molecules (PD-1^hi^, TIM3^hi^) and exhaustion marker genes (*HAVCR2*, *TOX*) (11, 40), contained cells with high clonal expansion and correlated with the stimulated CD4_Stim2.Tex cluster expressing *IFNG* and *IL21* (Figure 4, J–L, and Supplemental Figure 5, B–F). The CD4_Tact2 cluster contained CD4^+^ T cells with high relative expression of type-I IFN-inducible genes (*IFIT3*, *MX1*, and *HERC5*) (Supplemental Figure 5B) (56).

Cells within the CD4_Tfh cluster expressed relatively lower level of CD39 and shared TCRs with effector cells within several clusters (CD4_Tex, CD4_Th17-like, CD4_pTreg, CD4_Treg) (Figure 4D and Supplemental Figure 6, A–C). These results suggest that the CD4_Tfh cluster could contain a transitional population that gives rise to more differentiated effector CD4^+^ T cell populations in tumors. Through GSEA, we did not observe any significant correlation of CD4_Tfh, CD4_Th17-like, or CD4_Tex gene sets with clinical outcomes (Supplemental Figure 6, D and E).

### CD38^+^ pTreg infiltrates correlate with poor clinical outcomes in CRC.

**Figure 5:**
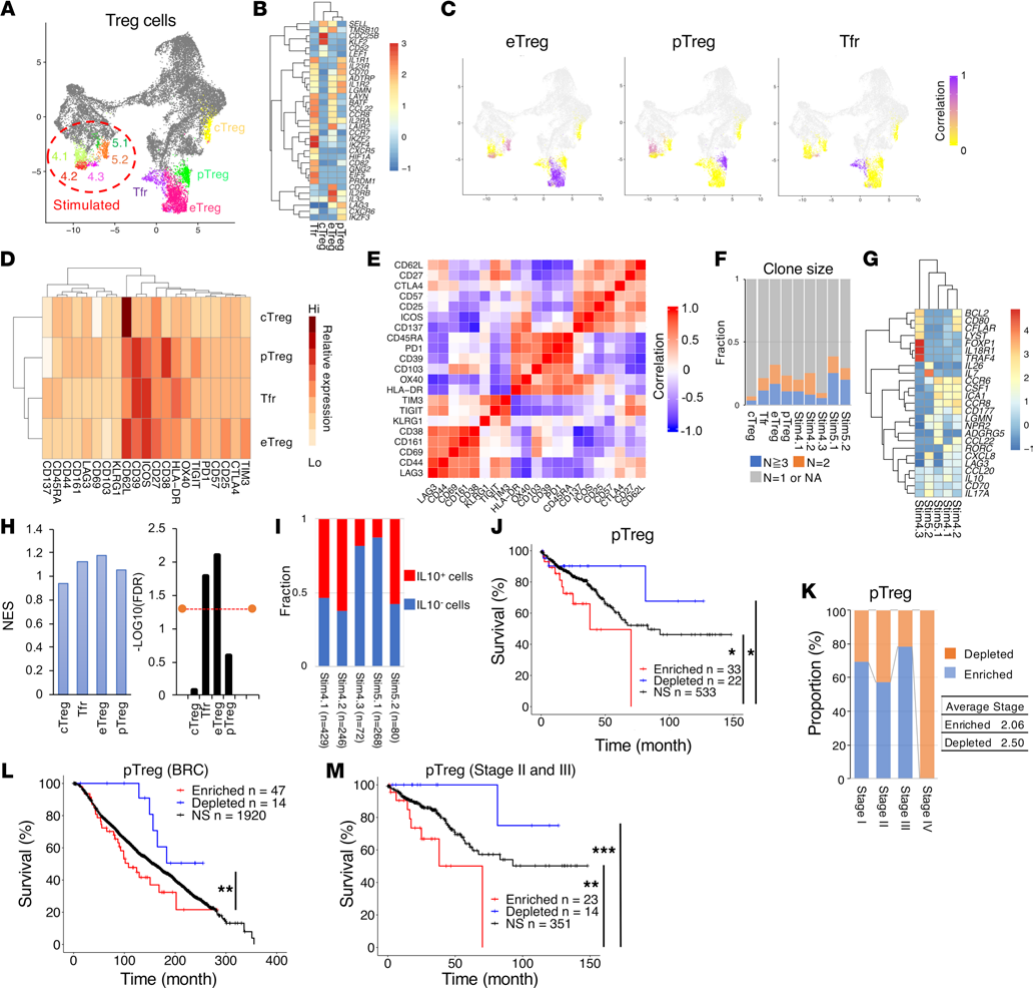
Treg subpopulations with opposite clinical outcomes in CRC. (**A**) Treg subpopulations on the CD4 UMAP from Figure 4A. (**B**) Heatmap of differentially expressed genes (as indicated) within Treg subpopulations. (**C**) Heatmap of Pearson correlation between nonstimulated and stimulated cells within each Treg subcluster, correlated based on 21 ADT signals. (**D**) Heatmap depicting average ADT signal in the CITE-Seq antibody panel within each nonstimulated Treg subpopulation. (**E**) Heatmap showing Pearson correlations between 21 ADT signals from each nonstimulated Treg subcluster. (**F**) Bar graph depicting clonal composition of T cells within each Treg subpopulation. (**G**) Heatmap of differentially expressed genes in stimulated Treg subclusters. (**H**) NES and FDR results from GSEA between the leading-edge genes (in Supplemental Figure 7D) of the positively prognostic T_Treg cluster (in Figure 2C) and signature genes in each Treg subpopulation. FDR threshold value indicated by the orange line is 0.05. (**I**) Bar graphs depicting proportion of intratumoral *IL10*^+^ and *IL10*^–^ Tregs within each stimulated Treg subcluster. The number of cells within each cluster were indicated. (**J**, **L**, and **M**) Kaplan-Meier curves of overall survival in TCGA cohorts for patients enriched or depleted for the following gene sets using GSEA (Methods): pTreg (CRC) (**J**), pTreg cluster (BRC) (**L**), or pTreg (CRC, Stage II and Stage III) (**M**). *P < 0.05; **P < 0.02; ***P < 0.001 (log-rank test). See detailed *P* values (Supplemental Table 6). (**K**) Bar graphs depicting relative proportion of patients by stage enriched or depleted for CD4_pTreg gene sets in CRC. Average stage is indicated on right.

To investigate their functional capacity, Treg populations were further subdivided by unsupervised clustering, resulting in 4 distinct nonstimulated and 5 distinct stimulated subpopulations (Figure 5A). Based on the signature genes, we designated nonstimulated subpopulations cTreg (central Treg), pTreg, eTreg (effector Treg), and Tfr (follicular Treg) (Figure 5B and Supplemental Figure 7A). Based on 21 ADT signals, we were able to link these Treg subpopulations with cognate subpopulations within stimulated subclusters (Stim4.1, Stim4.2, and Stim4.3 within Stim4 cluster; Stim5.1 and Stim5.2 within Stim5 cluster) (Figure 5C).

Helios^+^ Tregs within the CD4_Treg cluster were subdivided into the CD4_eTreg and CD4_Tfr clusters (Figure 5A). The CD4_eTreg cluster, containing cells highly expressing CD62L, ICOS, and CD25 with high clonal expansion (>30%), correlated with its cognate-stimulated subclusters (CD4_Stim4.2 and CD4_Stim5.1), which contained cells with expression of genes related to trafficking (*CCR6*, *CCL22*) (Figure 5, D–F). The CD4_Tfr cluster was phenotypically similar to the CD4_eTreg but distinguishable by the Tfr marker gene *CXCR5* (Supplemental Figure 7A). Tfr cells also expressed the *PDLM1*, encoding the transcription factor Blimp-1, a marker of eTregs in mice (57), and had high relative expression of OX40 and HLA-DR (Figure 5, B and D). The CD4_Tfr cluster correlated with cognate-stimulated cluster CD4_Stim4.3, expressing genes (*IL18R1*, *FOXP1*, *BCL2*, *TRAF4*) related to the activation and maintenance of Tregs (Figure 5G) (58–61). Although neither CD4_eTreg nor CD4_Tfr gene sets were associated with a favorable prognosis in CRC, leading-edge genes within the positively prognostic T_Treg cluster were relatively enriched in eTreg and Tfr cells compared with pTregs (Figure 5H and Supplemental Figure 7, B–D). eTregs had significant TCR sharing with Tfr cells but limited sharing with pTregs, suggesting they share ancestry and originate independently of pTregs (*P* < 0.0001; Supplemental Figure 7E). Thus, our results suggest that Helios^+^ Tregs, as opposed to Helios^–^ Tregs, account for the majority of total Tregs associated with favorable outcomes in CRC.

The CD4_pTreg cluster was linked to cognate-stimulated clusters CD4_Stim4.1 and CD4_Stim5.2, which expressed Th17 signature genes *IL17A* and/or *RORC* and a higher level of *IL10* than other Treg clusters (Figure 5, G and I). Through GSEA, CD4_pTreg signature genes were enriched in both the CRC and BRC cohorts with poor outcomes (Figure 5, J–L). Moreover, CRC patients with Stage II and Stage III tumors and enrichment of the CD4_pTreg gene set strongly correlated with a poor prognosis, compared with those of depletion (*P* < 0.01), indicating that enrichment of pTregs was not reflective of stage (Figure 5M). Interestingly, there was substantial overlap between CD4_pTreg-enriched and CD4_Th17-like–enriched patients within the CRC cohort (76%; 25 of 33 enriched patients) but no overlap between CD4_pTreg-enriched and CD4_Th17-like–depleted patients (Supplemental Figure 7F). In addition, cells within both CD4_pTreg and CD4_Th17-like clusters shared T cell clones, although TCR sharing was not confined to these clusters (Supplemental Figure 7G). These results indicate that pTregs and Th17 cells in CRC are functionally and phenotypically linked and enriched in the same patients. We performed IHC on CRC specimens derived from different patients to validate distinct Treg subpopulations we found. We confirmed the presence of both CD38^+^ and CD38^–^ FOXP3^+^ Tregs within different patients (Supplemental Figure 7, H and I). Taken together, these data demonstrate substantial phenotypic and functional heterogeneity of Tregs within CRC and indicate that distinct Treg populations associate with opposing clinical outcomes.

## Discussion

Prior studies performed through histology or bulk RNA-Seq analysis demonstrated a relationship between T cell infiltrates and clinical outcomes in CRC (2, 3, 9, 12, 57). These studies were inherently limited by their low dimensionality, as a relatively limited number of marker genes was assessed in these studies, thus hampering the identification of distinct T cell subtypes with high granularity and leading to their conflicting associations with clinical prognosis. Here, our single-cell analysis enabled a highly granular assessment of diverse CD8^+^ and CD4^+^ T cell subtypes in CRC. Our data demonstrate high diversity and heterogeneity of T cells within tumors, which account for the discrepancies reported in prior bulk analyses. Moreover, our statistical analysis using GSEA enabled us to link these T cell subsets identified by single-cell analysis to patient prognosis within the large TCGA cohorts. We were able to independently corroborate findings from other studies, including the positive prognostic significance of Tregs and *GZMK*^+^ effector memory T cells in CRC (4, 14, 21). We were also able to uncover several potentially novel prognostic insights into distinct T cell subsets with potential therapeutic implications.

Of CD8^+^ T infiltrates in CRC, we found that 2 distinct cytotoxic T cell types differentiated into different types of clonally expanded Tex cells with coexpression of CD39 and CD103, consistent with tumor reactivity (34, 35). One of the cytotoxic T cell types, which exhibited a Tem cell phenotype and expressed the naive T cell marker *TCF7*, differentiated into a Tex subpopulation with a less dysfunctional transcriptional state (*GZMA*, *GZMK*, *PRF1*). These Tem cells and Tex subpopulation accounted for the majority of the positively prognostic T cells (within the T_Tcyto1 cluster), suggesting that these Tem cells — which are able to produce cytokines including *IFNG*, *IL-2*, and *TNF* — may possess antitumor activity. In contrast, *GNLY*^+^CD103^+^ T cells, predominantly consisting of IELs (within the T_Tcyto2 cluster), differentiated into a Tex subpopulation with a more dysfunctional phenotype (PD-1^+^, TIM3^+^) associated with loss of cytokine production as described above. Of note, this Tex population was present within tumors but almost absent in normal colon and expressed relatively higher levels of CD39 and CD103 than the Tex population originating from Tem cells. Nonetheless, this cluster did not correlate with positive clinical outcomes in CRC, although it did in melanoma. This discrepancy between CRC and melanoma may reflect disparate functions of cells with T_Tcyto2 gene signature in these 2 cancers. Interestingly, Trm infiltrates have been shown to correlate with positive outcomes in several tumor types, including melanoma (37, 62), with the notable exception of CRC. One study has reported CD103^+^ T cell infiltrates to be associated with poor outcomes in CRC (63). The presumptive function of these tumor-reactive cells is through cell-autonomous antitumor activity. Taken together, our data suggest that tumor-reactive cytotoxic T cells may indeed be properly targeted to the tumor, but that in itself may not serve in controlling CRC. Indeed, our data suggest that their presence alone is insufficient to predict their effectiveness but rather highlights the importance of their cell-autonomous functional properties. The discordance we found between presumed tumor reactivity and prognostic significance of the Trm phenotype T cells in CRC suggests that the factors regulating dysfunction in these cells in CRC may be potential targets for therapy.

Compared with CD8^+^ T cell infiltrates, CD4^+^ T cell infiltrates within tumors exhibited more complexity and diversity. Interestingly, both total T cell cytotoxic clusters (T_Tcyto1 and T_Tcyto2) contained CD4^+^ cytotoxic T cell types marked by *CCL5* expression, respectively, while CD4_Tcyto2 cells were not confined to tumors. Although a developmental link between those cells and CD4 exhausted cell types was not observed, CD4^+^ T cells (CD4 T_cyto1) within the positively prognostic T_Tcyto1 cluster expressed relatively higher expression of effector molecules and coinhibitory molecules such as PD-1, indicating that they may be important in immunotherapy, as has been shown for cytotoxic *GZMK*^+^CD4^+^ T cells with a similar gene signature in bladder cancer (64). Further investigation is warranted to ascertain a role of this cytotoxic CD4 cell type in CRC.

Although T-bet^+^ T cell infiltration has been associated with a favorable prognosis in CRC (3, 4), our single-cell analysis showed substantial diversity in CRC, with expression of T-bet observed not only in Th1 cells, but also in Th17 cells and Tregs. While such T-bet^+^ effector T subtypes may be therapeutically important, we could not find differences in outcomes between gene set-enriched or -depleted patients from bulk-Seq data of the TCGA cohort, possibly due to heterogeneity of Th1-infiltrates within tumors.

Of T cell infiltrates in CRC, *CXCL13*^+^CD4^+^ T cells — including Tfh and CD4^+^ Tex cells — expressed the highest levels of PD-1. Interestingly, MSI-H patients within the TCGA were preferentially enriched with CD4^+^ Tex gene signatures (53 %; 16 of 30 CD4^+^ Tex–enriched patients). This result affirms findings from a prior study that showed that Th1-like cells were enriched in MSI-H patients, and it suggests that they may mediate responses to immunotherapy (11). Our study further identified that Tfh cells with a more naive phenotype were developmentally linked to CD4^+^ Tex cells through TCR sharing, suggesting that these Tfh cells can differentiate into CD4^+^ Tex cells in CRC.

With respect to *FOXP3*^+^ T cells (Tregs), our data show that all Treg subpopulations were relatively enriched within tumors and expressed the activation markers CD39 and ICOS. In those Tregs, we found 2 phenotypically and functionally distinct Treg infiltrates in CRC, Helios^+^ Tregs and Helios^–^ Tregs (pTregs), which arise independently based on TCR sharing. Interestingly, these Tregs were associated with opposite clinical outcomes. Total Tregs, of which Helios^+^ Tregs accounted for the majority, were enriched in patients with early-stage cancer and improved outcomes, consistent with the preponderance of prior studies of Tregs in CRC (21, 22). In contrast, enrichment of pTregs strongly correlated with poor clinical outcomes in a stage-independent manner. While mechanisms dictating these differences in clinical outcomes warrant further investigation, pTregs but not Helios^+^ Tregs highly expressed CD38 and LAG3 and were capable of producing higher levels of IL-10, suggestive of immunosuppressive activity. pTregs also expressed Th17 signature genes, including *IL17A*, *RORC*, *IKZF3,* or *IL23R*. In line with our data, *RORC*^+^*FOXP3*^+^ T cells were shown to suppress antitumor immunity in a mouse model of CRC (15). Thus, our data implicate CD38^+^ pTregs as the population that suppresses antitumor immunity in CRC and a target of immunotherapy. Intriguingly, daratumumab, an anti–human CD38 monoclonal antibody used to treat myeloma, has been shown to target CD38-expressing Tregs (65).

## Methods

### Human colon biopsy and single-cell dissociation.

Tumors and adjacent normal colon tissues from the patients, diagnosed with colorectal adenocarcinoma at Columbia University Irving Medical Center (CUIMC), were collected with informed consent. All protocols were performed in accordance with the guidelines provided by the IRB. All patients were treatment naive at time of sample acquisition (Supplemental Table 1). Fresh tissue was collected the same day of surgery. Tissue was dissociated into single-cell suspensions with enzymic digestions using a gentleMACS dissociate per protocol (Miltenyi Biotec). Single-cell suspensions were cryopreserved (10% DMSO) and stored in a liquid nitrogen.

### IHC.

Samples that had been fixed in formalin were washed in 70% ethanol and subjected to standard dehydration processing to prepare them for mounting in paraffin wax blocks (Supplemental Table 10). These paraffin blocks were cut into 5 μm sections. Following deparaffinization, antigen-retrieval was performed in citrate buffer, pH 6.0 (Vector laboratories, H-3300). Blocking was performed in tris-buffered saline with normal goat serum 10% v/v, 1%BSA (Roche, 10735086001. Slides were incubated overnight at 4°C with anti–human FoxP3/IgG2a monoclonal (PCH101) rat antibodies (10 μg/mL) (eBioscience from Thermo Fisher Scientific, catalog 14-4776-82), anti–human CD38/IgG1 monoclonal (HIT2) mouse antibodies (10 μg/mL) (eBioscience from Thermo Fisher Scientific, catalog 14-0389-82), anti–human CD4/IgG monoclonal (SP35) rabbit antibodies (1:50 dilution) (Abcam, catalog ab213215), anti-CD8/IgG2b monoclonal (YTC182.20) rat antibody (20 μg/mL) (Invitrogen from Thermo Fisher Scientific, catalog MA1-81692), anti-CD57/IgM monoclonal (HNK-1) mouse antibody (1:100 dilution) (Cell Signaling Technology, catalog 72031), and anti-CD103/IgG1 monoclonal (LF61) mouse antibody (20 μg/mL) (Invitrogen from Thermo Fisher Scientific, catalog MA1-33553). Secondary antibodies were incubated 1 hour at room temperature followed by DAPI (Sigma-Aldrich, 10236276001) counterstaining. Fluorescence imaging was performed with Leica DMi8 microscope (Leica Microsystems).

### Antibody-oligo conjugates.

Antibodies for CITE-Seq and Cell Hashing were purchased from BioLegend and were covalently conjugated to barcoded oligos by click-chemistry tools in accordance with the protocol of CITE-Seq Hyper Antibody-Oligo Conjugation (https://cite-Seq.com/protocol/). The list of antibodies, clones, and barcodes are available in Supplemental Table 2.

*T cell isolation, T cell stimulation, and cell sorting from CRC and adjacent normaltissues*. Cryopreserved single-cell suspensions were thawed at 37°C, and single T cells were isolated by percoll (MilliporeSigma, GE17-0891-01) density gradient (20%/40%/80%) centrifugation (400*g* for 30 minutes at 4°C). T cells were rested in complete RPMI in 96-well plates overnight. The next day, T cells from each patient were split into 2 wells, incubated with or without PMA/Ionomycin (Thermo Fisher Scientific, 00-4970-03). After 3 washes, cells were incubated for 10 minutes with Fc receptor block (BioLegend, 422302) to block nonspecific antibody binding; each sample was stained with Cell Hashing antibody cocktail for 30 minutes. After 3 washes, cells were stained with CITE-Seq antibodies and FACS antibody cocktails for 30 minutes (Supplemental Table 2). Viable T cells were sorted by FACS Aria II by gating DAPI^–^ZombiAqua^–^CD3^+^TCRαβ^+^ T cells (Supplemental Figure 1A).

*Single cell 5′transcript, TCRαβenriched, and Cell Hashing/CITE-Seq library preparation on the 10× Chromium, and sequencing*. The scRNA-Seq, scTCR-Seq and HTO/ADT-Seq libraries were prepared using the 10× Chromium single-cell 5′ library and gel bead kit, per manufacturer’s instructions. In brief, oligo-conjugated antibody-tagged single-cell suspensions were loaded into the Chromium controller to make nanoliter-scale droplets with uniquely barcoded 5′ gel beads called GEMs (gel bead-in emulsions). After GEM-RT, GEMs were cleaned up by Dyna beads MyOne Silane beads (Thermo Fisher Scientific, 37002D). At the cDNA amplification step, HTO/ADT additive oligos were added to amplify oligos derived from Cell Hashing and CITE-Seq antibodies. The products were size separated with SPRI Beads (Beckman Coulter, B23317): < 300 nt fragments containing the oligos derived from CITE-Seq antibodies and Cell Hashing, and > 300 nt fragments containing cDNAs derived from cellular mRNA. HTO/ADTs were amplified using specific primers that append P5 (5′- AATGATACGGCGACCACCGAGATCTACAC -3′) and P7 (5′- CAAGCAGAAGACGGCATACGAGAT -3′) sequences for Illumina sequencing.

The cDNA was then pooled for downstream processing and library preparation according to the manufacturer’s instructions. The 5′ transcript library was sequenced with Illumina NovaSeq. The HTO/ADT libraries were sequenced with Illumina MiSeq or NextSeq. The single cell TCR enriched library was sequenced with Illumina MiSeq using 150 paired-end reads.

### Data processing of HTO/ADT libraries.

We counted ADTs or Cell Hashing tags (HTOs) in raw sequencing reads and built a count matrix using CITE-Seq-Count as previously described, available at https://hoohm.github.io/CITE-seq-Count/ For HTO quantification, each HTO count was normalized by total counts of HTOs per cell barcode (total UMI counts were set at 100). Then, cell barcode identity was assigned to the most highly expressed HTO, otherwise assigned to “negative” or “doublet”, available at https://github.com/akornberg/data_analysis_ak Each CITE-Seq UMI count was normalized by the average of total UMI counts from all cell barcodes; then, a count matrix was set into the Seurat as the ADT data, which was normalized based on the Seurat package, CLR normalization, and scaled.

### Data processing of scRNA-Seq libraries.

The total reads from scRNA-Seq were aligned to GRCh38 reference genome and quantified using cellranger count (10× Genomics, version 3.1.0). In brief, only cells that passed the threshold set by the pipeline (more than 500 UMIs) were considered for further analysis. On average, we obtained reads from 1862 genes per cell (median, 1572) and 4908 unique transcripts per cell (median, 4178). The analysis of the created filtered feature-barcode matrices was performed using Seurat (version 3.1.3), available at https://satijalab.org/seurat/

### Data processing of single-cell TCR-Seq libraries.

The total reads from scRNA-Seq were aligned to GRCh38 reference genome and consensus TCR annotation was conducted by cellranger vdj (10× Genomics, version 2.1.0). In total, 86% (27,999 of 32,550 cells) of annotated T cells were assigned a TCR (TRB and/or TRA), and 20,875 clonotypes were detected.

### Dimension reduction and clustering.

For the downstream analysis, 4 multiple scRNA-Seq data sets (Seurat objects) with different individuals and different conditions (Supplemental Table 3) were created by the Seurat, in which the metadata for HTO assignment (patients, stimulated cells, or nonstimulated cells), normalized ADT counts (23 CITE-Seq antibodies), and TCR clonotypes and sequences were combined into the data sets using the function AddMetadata and CreateSeuratobject, and they were integrated using an anchor-based single-cell data integration method in Seurat (66). In the method, the batch effects among the samples in 4 different data sets were normalized using the function SCtransform in Seurat. Cells with mitochondrial RNA content greater than 10% were excluded. Variable feature genes were set at 5000 genes. Once the data sets were integrated, the data were input into a principal component analysis (PCA) on the basis of variable genes. The same principal components were used to generate the Uniform Manifold Approximation and Projections (UMAPs). Clusters were identified using shared nearest neighbor–based (SNN-based) clustering. For the clustering analysis, the function RunUMAP, FindClusters, and FindNeighbors in Seurat were used, in which “dims” or “resolution” were set at between 10 and 30 or between 0.1 and 0.5, respectively.

### Analysis of TCR sharing and clone frequency.

Identical TCR clonotypes (regarded as clonal identifier) among single ells were counted through the Seurat metadata. Cells missing an identifier were considered as not observed (shown as NA). TCR sharing between 2 distinct clusters or different cell states on the UMAP were assessed by the counts of identical TCRβ clonotypes. After CD4^+^/CD8^+^ single-cell separation, clone frequency in each single cell was calculated as a relative value of TCRβ clonotype counts to total single cells from each patient (TILs were separated from normal cells). Code is available at https://github.com/akornberg/data_analysis_ak

### Gene-to-gene correlation analysis and gene signature scoring.

The stem-like, exhausted, and cytotoxic signature genes were selected from variable genes across all CD8^+^ T cells, which were computed using the Seurat. Those signature genes consisted of the top 20–30 genes with the highest correlation with the reference genes *TCF7*, *HAVCR2*, or *FGFBP2* as previously described (42) (Supplemental Table 8). Individual cells on the CD8 UMAP (nonstimulated) were scored by the average of the selected gene expression and shown as dot plots.

### GSEA using TCGA cohort.

Gene expression data of the TCGA cohort were obtained from cBioPortal (https://www.cbioportal.org/). The TCGA cohort data of CRC, BRC, and melanoma were downloaded from Colorectal Adenocarcinoma (TCGA, PanCancer Atlas), Breast Cancer (METABRIC), and Skin Cutaneous Melanoma (67, 68) (TCGA, PanCancer Atlas). Gene expression was normalized by log_2_ transformation, and an average *Z* score across patient samples was calculated per gene. For each patient, genes were ranked by the average *Z* score in each gene. Regarding original gene sets in distinct T cell subsets delineated by unsupervised clustering, genes were ranked by differential expression test, which was carried out using Seurat. The top genes (~100 genes) were selected with a Seurat function “FindMarkers” in which min.diff.pct was set at > 0.25 after removing specific noncoding RNA such as long intergenic noncoding RNA (lincRNA) and miRNA and genes linked with poorly supported transcriptional models (annotated with the prefix “AP-”) (Supplemental Table 5). The correlation between the gene sets and bulk databases of the TCGA cohort was calculated using preranked GSEA method fgsea package (https://github.com/ctlab/fgsea).

### Prognosis prediction.

The CRC, melanoma, and BRC TCGA cohort were grouped on the basis of GSEA. One group (normalized enrichment score [NES] > 0 and a Benjamini-Hochberg–adjusted [BH-adjusted] *P* < 0.05) was categorized into “Enriched”. The other (NES < 0 and BH-adjusted *P* < 0.05) was assigned into “Depleted”. Kaplan-Meier survival curves were generated, and log-rank test for 2 groups was performed using the R package Survival (3.1.8).

### Diffusion map trajectory analysis.

The Seurat object including the clusters CD8_Tn/Tcm, CD8_Trm, CD8_Tem, CD8_Temra, CD8_IEL, and CD8_Tex, which were characterized by unsupervised clustering in Seurat (Figure 3A), were subjected into single-cell trajectory analysis using the Monocle (2.14.0), available at (http://cole-trapnell-lab.github.io/monocle-release/). RNA expression data from the Seurat object were converted into Monocle as a sparse matrix. The metadata from the Seurat object (total single cells in each patient, as well as cluster annotation) were incorporated as “phenodata” in Monocle. Then, newCellDataSet was created with a function “expressionFamily = negbinomial.size()”. In order to infer a single-cell trajectory, the total 650 genes (Supplemental Table 9) that were assembled from each cluster in the Seurat object using differential expression test were chosen; then, the cells were ordered after dimension reduction with “DDR-tree” function and visualized.

### Gene or ADT expression heatmap and correlation analysis.

For single-cell gene heatmaps, the representative marker genes were selected from differentially expressed genes within each cluster that was identified by unsupervised clustering. Gene heatmaps between cells within each cluster were created using the function “DoHeatmap” in Seurat (Supplemental Figure 4B and Supplemental Figure 5B).

For ADT heatmaps and gene heatmaps among subclusters, each average ADT signal of cells within each cluster or average expression of each differentially expressed gene in each subcluster was calculated using the function “AverageExpression” in Seurat. Then, a heatmap of each average ADT signal or gene expression was shown in selected clusters using the R package pheatmap (1.0.12).

The relationship between stimulated and nonstimulated clusters was assessed by a statistical correlation between gene expression or average ADT signals in single cells. In brief, for ADT correlation analysis, a matrix including each average ADT signal in stimulated and nonstimulated clusters was created, and its correlation was examined by Pearson correlations. For gene expression correlation analysis, the representative marker genes were selected from differentially expressed genes within each nonstimulated cluster, and a matrix including each average variable gene expression in each stimulated cluster was created. Then, a correlation between marker genes in nonstimulated clusters and average gene expression in stimulated clusters was examined by GSEA using the R package fgsea.

### Data and materials availability.

The accession numbers for the sequencing results reported in this paper are ArrayExpress: E-MTAB-9455. Fastq files from each sequencing lane (L1, L2) and corresponding to each fragment end (R1, R2) were concatenated into a single file to generate 2 fastq files (R1, R2) per biological replicate. Code is available at the links as indicated previously in Methods.

### Statistics.

GSEA and prognosis prediction were described in above. Log-rank test was used for comparison of the survival distribution between gene-enriched, gene-depleted, or nonenriched patients form GSEA analysis. Wilcoxon rank-sum test was used for all nonpaired comparisons. A P value less than 0.05 was considered significant.

### Study approval.

This study was approved by the Columbia University IRB. Written informed consent was obtained from each patient.

## Author contributions

Study concept and design was contributed by KM and AH. Patient and sample acquisition was contributed by KM, SL, MI, VR, AMA, SAL, RPK, ADP, KSY, PEO, and AH. Analysis of data was contributed by KM and AH. Experiments were designed and performed by KM, SL, JM, TB, LC, KAS, MS, PS, PAS, and KSY. Computational analysis was contributed by KM, AK, TB, AMB, and PAS. KM, NS, KSY, and AH wrote the manuscript. Study oversight was contributed by AH.

## Supplementary Material

Supplemental data

## Version 1 02/22/2022

In-Press Preview

## Version 2 04/08/2022

Electronic publication
