# Supplementary material for: Multiplexed single-cell analysis reveals prognostic and nonprognostic T cell types in human colorectal cancer
Source: JCI Insight. 2022 Apr 8;7(7):e154646. doi: 10.1172/jci.insight.154646 (PMC9057629; doi:10.1172/jci.insight.154646)
Supplement: Supplemental data [file jciinsight-7-154646-s186.pdf]

Supplemental material for:

Multiplexed single-cell analysis reveals prognostic and non-prognostic T cell types in human colorectal cancer

Kazuya Masuda[1], Adam Kornberg[1,2], Jonathan Miller[3,4], Sijie Lin[1], Nathan Suek[1], Theo Botella[4], Kerim Secener[1], Alyssa M. Bacarella[3], Liang Cheng[4], Matthew Ingham[5,6], Vilma Rosario[6,7], Ahmed M. Al-Mazrou[6,7], Steven A. Lee-Kong[6,7], Ravi P. Kiran[6,7], Marlon Stoeckius[9], Peter Smibert[9], Armando Del Portillo[10], Paul E. Oberstein[5,6], Peter A. Sims[12], Kelley S. Yan[8,11], Arnold Han[,1,2,6,8*]

[1]Columbia Center for Translational Immunology, Columbia University, New York, NY

[2]Department of Microbiology & Immunology, Columbia University, New York, NY

[3]Department of Pediatrics, Columbia University, New York, NY

[4]Columbia Center for Human Development, Columbia University, New York, NY

[5]Department of Medicine, Division of Hematology & Oncology, Columbia University, New York, NY

[6]Herbert Irving Comprehensive Cancer Center, Columbia University, New York, NY

[7]Department of Surgery, Division of Colorectal Surgery, Columbia University, New York, NY

[8]Department of Medicine, Division of Digestive & Liver Diseases, Columbia University, New York, NY

[9]New York Genome Center, New York, NY

[10]Department of Pathology, Columbia University, New York, NY

[11]Department of Genetics & Development, Columbia University, New York, NY

[12]Departments of Systems Biology and Biochemistry & Molecular Biophysics, Columbia University, New York, NY

Conflict of interest: The authors have declared that no conflict of interest exists.

*Correspondence: Arnold Han; Address: Columbia University, BB 15-1501E, 650 W. 168th St., New York, NY 10032; Email: ash3@cumc.columbia.edu; Phone: +1-212-305-2137

Supplemental Table

Supplemental Table 1. Patient nomenclature

Supplemental Table 2. Antibody clones and barcodes for CITE-seq and cell hashing

Supplemental Table 3. Experimental datasets for scRNA-seq, TCR-seq, and CITE-seq

Supplemental Table 4. Cluster cell number by patient and sample

Supplemental Table 5. Gene sets for GSEA (related to Figure 2 and Supplemental Figure 3)

Supplemental Table 6. P-values for Kaplan Meier analysis (related to Figure 2 and 5 and Supplemental Figure 3, 6, and 7)

Supplemental Table 7. MAIT cell distribution in the CD8 UMAP

Supplemental Table 8. Gene lists highly correlated with marker genes *TCF7*, *FGFBP2*, or *HAVCR2*

Supplemental Table 9. Gene lists for cell trajectory analysis (related to Figure 3I and Supplemental Figure 4F)

Supplemental Table 10. IHC patient characteristics

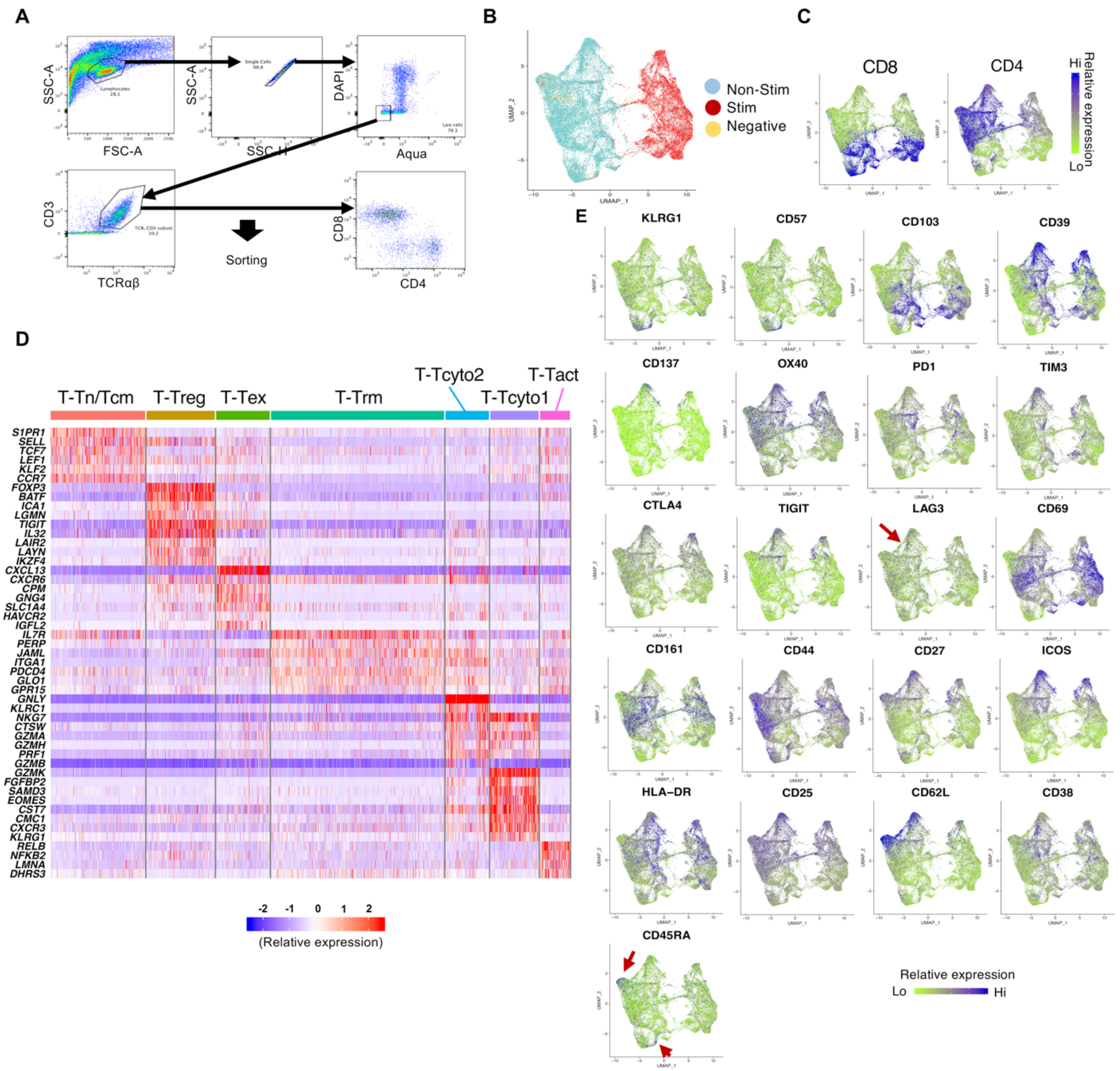

Supplemental Figure 1. Landscape of single T cells in CRC and adjacent normal colon characterized by its transcriptome and expression of 23 cell surface proteins. (**A**) Gating strategy for viable CD3^{+}TCRαβ^{+} T cell sorting from dissociated cells. Single lymphocytes were gated based on scatter. Viable CD3^{+}TCRαβ^{+} cells (DAPI negative, Aqua LIVE/DEAD negative) are sorted and single-cell sequencing was performed. Sorting strategy was validated through staining with fluorescently-labeled CD4/CD8 antibodies that do not compete with ADT clones. Data are representative of four independent experiments. (**B**) Distribution map of non-stimulated and stimulated cells on the total UMAP in Figure 1B. Unassigned cells are indicated in yellow. (**C**) Heatmap of CD4 or CD8 ADT signal on the total UMAP. Scale indicated on right. (**D**) Heatmap of differentially expressed genes between non-stimulated cells within each cluster (as indicated). (**E**) Each ADT signal in the CITE-seq antibody panel on the total UMAP. Arrows indicate cell populations expressing rare markers.

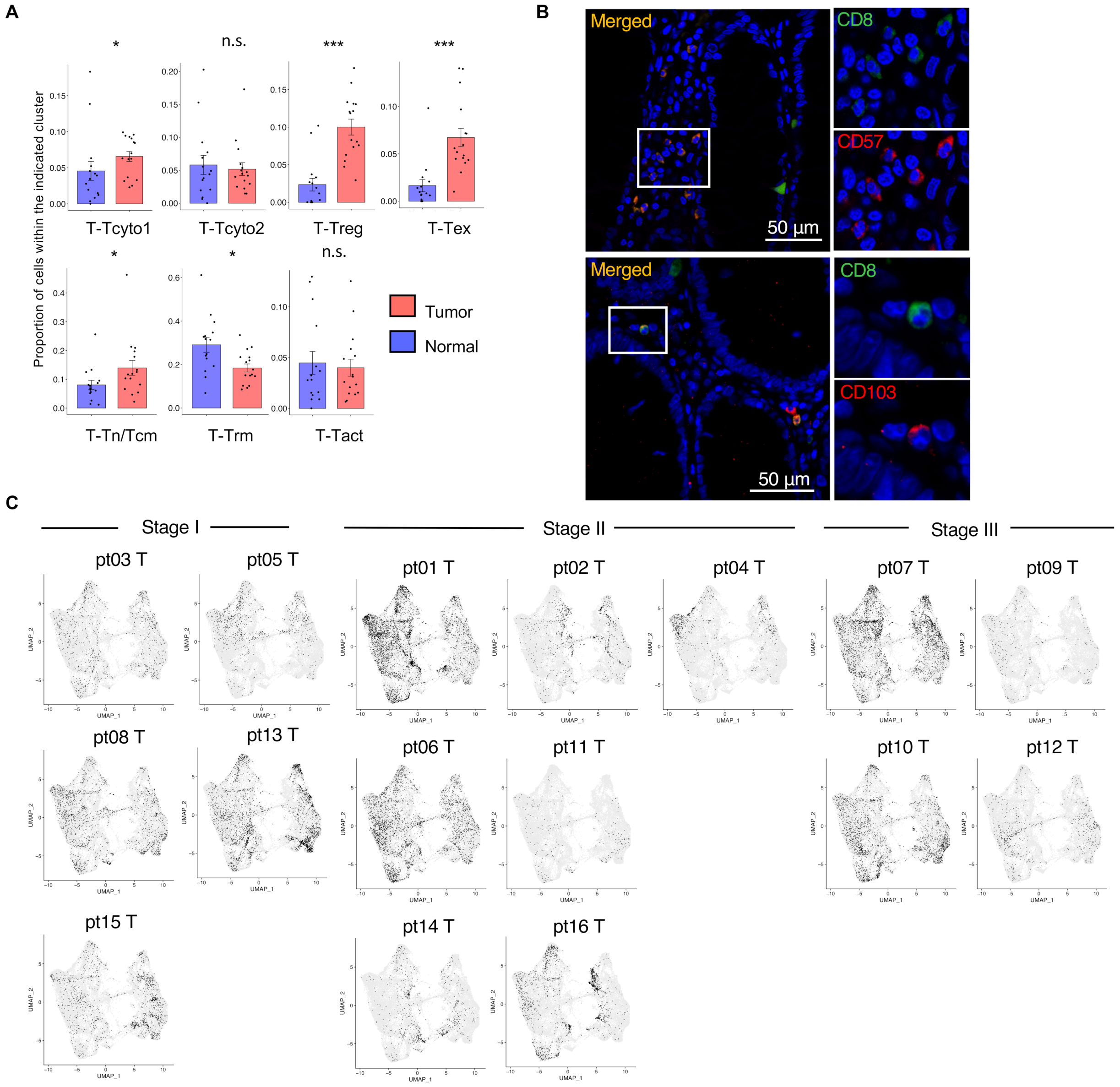

Supplemental Figure 2. Cell composition of each patient within tumors. (**A**) Proportion of intratumoral or normal cells within each cluster (as indicated) and its comparison. *** *P*-value (Wilcoxson test) <0.01; * *P* <0.05; n.s. (not significant) Pt 2 and Pt 10 were confirmed to have defects in mismatch repair (MSI-H) by IHC (**B**) IHC staining with anti-human CD8 and -CD57 antibodies or anti-human CD8 and -CD103 antibodies, respectively to validate target T cells in the T-Tcyto1 (upper) or the T-Tcyto2 cluster (lower) in stage 2/3 CRC (n = 2, exemplified by the patient A075 or A056, respectively, see Supplemental Table 10). (**C**) Distribution map of intratumoral cells in each patient by tumor-stage.

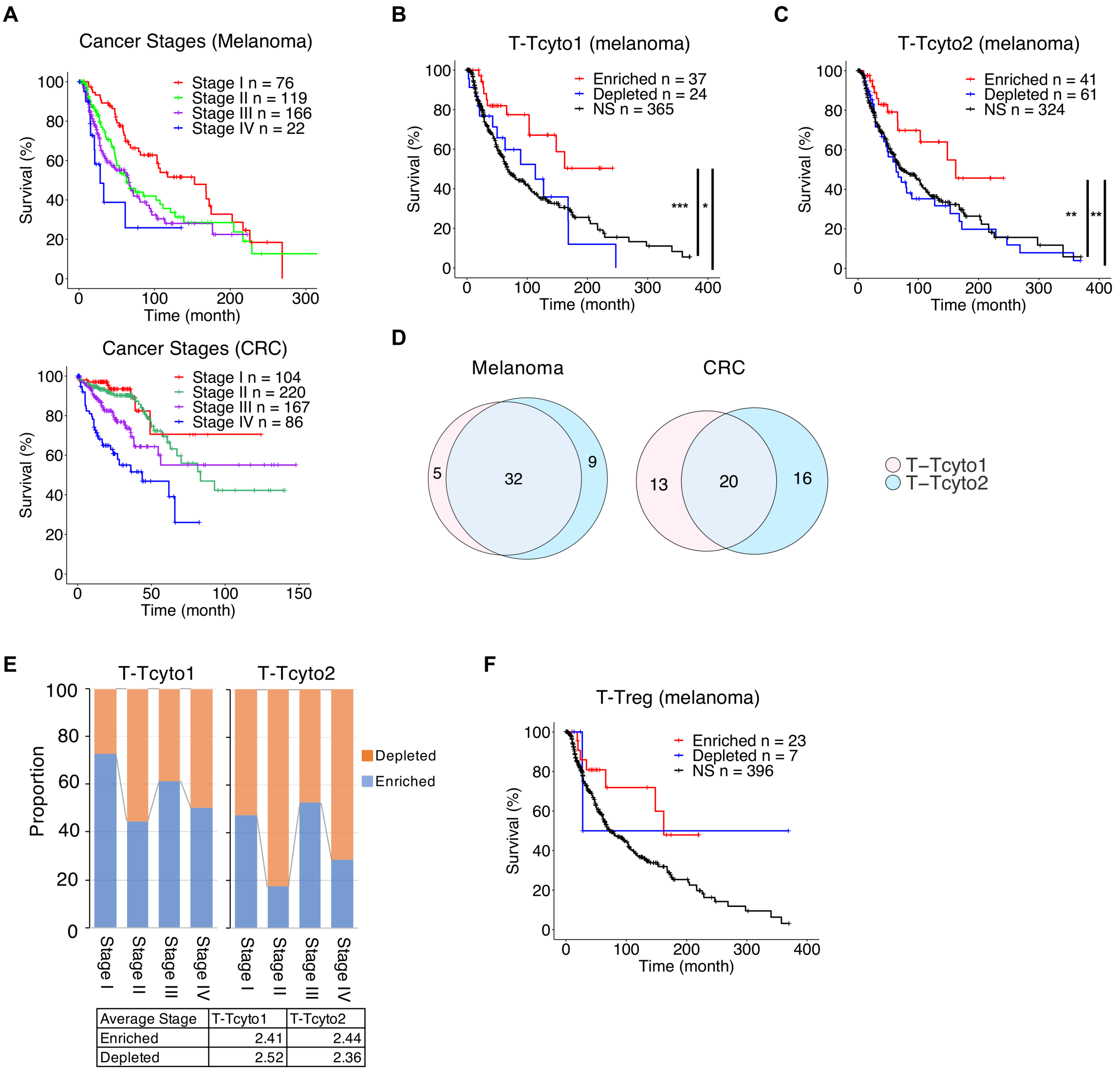

Average Stage	T-Tcyto1	T-Tcyto2
Enriched	2.41	2.44
Depleted	2.52	2.36

Supplemental Figure 3. Clinical outcomes associated with T cell subtypes identified by scRNA-seq in melanoma. (**A**) Kaplan-Meier curves of overall survival separated by clinical tumor-stage for the melanoma or CRC TCGA cohort. (**B, C** and **F**) Kaplan–Meier curves of overall survival in the melanoma TCGA cohort for patients enriched or depleted for the following gene sets using GSEA (Methods): T-Tcyto1 (**B**), T-Tcyto2 (**C**), or T-Treg cluster (**F**). * $P < 0.05$; ** $P < 0.02$; *** $P < 0.001$ (log-rank test). See detailed p-values (Supplemental Table 6). (**D**) Venn diagram showing the number of patients enriched for T-Tcyto1, T-Tcyto2, or both (center at light blue) in melanoma or CRC. (**E**) Bar graphs depicting relative proportion of patients by stage enriched or depleted for T-Tcyto1 (**B**), T-Tcyto2 (**C**). Average stage of patients is indicated in table on bottom.

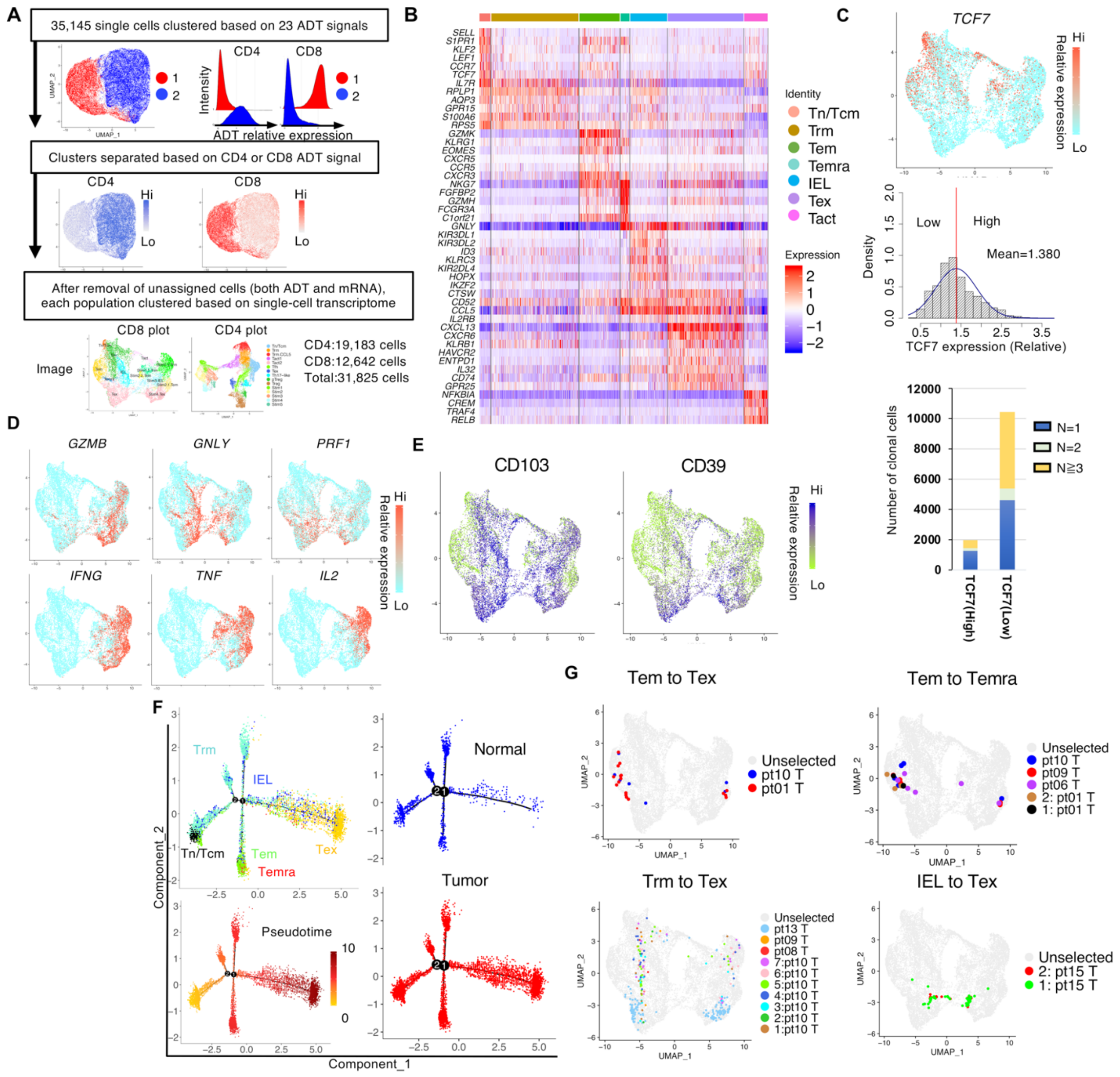

Supplemental Figure 4. Functional properties and relationships between distinct CD8⁺ T cell subtypes within tumors. (**A**) Workflow of CD8⁺/CD4⁺ T cell separation based on ADT signals and transcriptome from total 35,145 single cells. (**B**) Heatmap of differentially expressed genes (as indicated) between cells within each CD8 non-stimulated cluster. (**C**) Heatmap of the *TCF7* gene on the CD8 UMAP (top). *TCF7* mRNA expression levels are shown in density plot (middle). Bar blot depicts number of TCR clone cells with expression of high or low level of *TCF7* (separated by mean-value in density plot, bottom). (**D**) Heatmap of cytokine and effector genes (as indicated) on the CD8 UMAP. (**E**) Heatmap of CD103 or CD39 ADT signal on the CD8 UMAP. (**F**) Pseudotime diffusion maps depicting differentiation of non-stimulated CD8⁺ T cells. Cells were colored by each cluster (upper left) and pseudotime (lower left). Plots on right show normal or intratumoral cells from CD8 non-stimulated clusters, respectively. (**G**) TCR sharing between distinct cell states in Supplemental Figure 4F. TCR clone cells were investigated between distinct clusters as indicated.

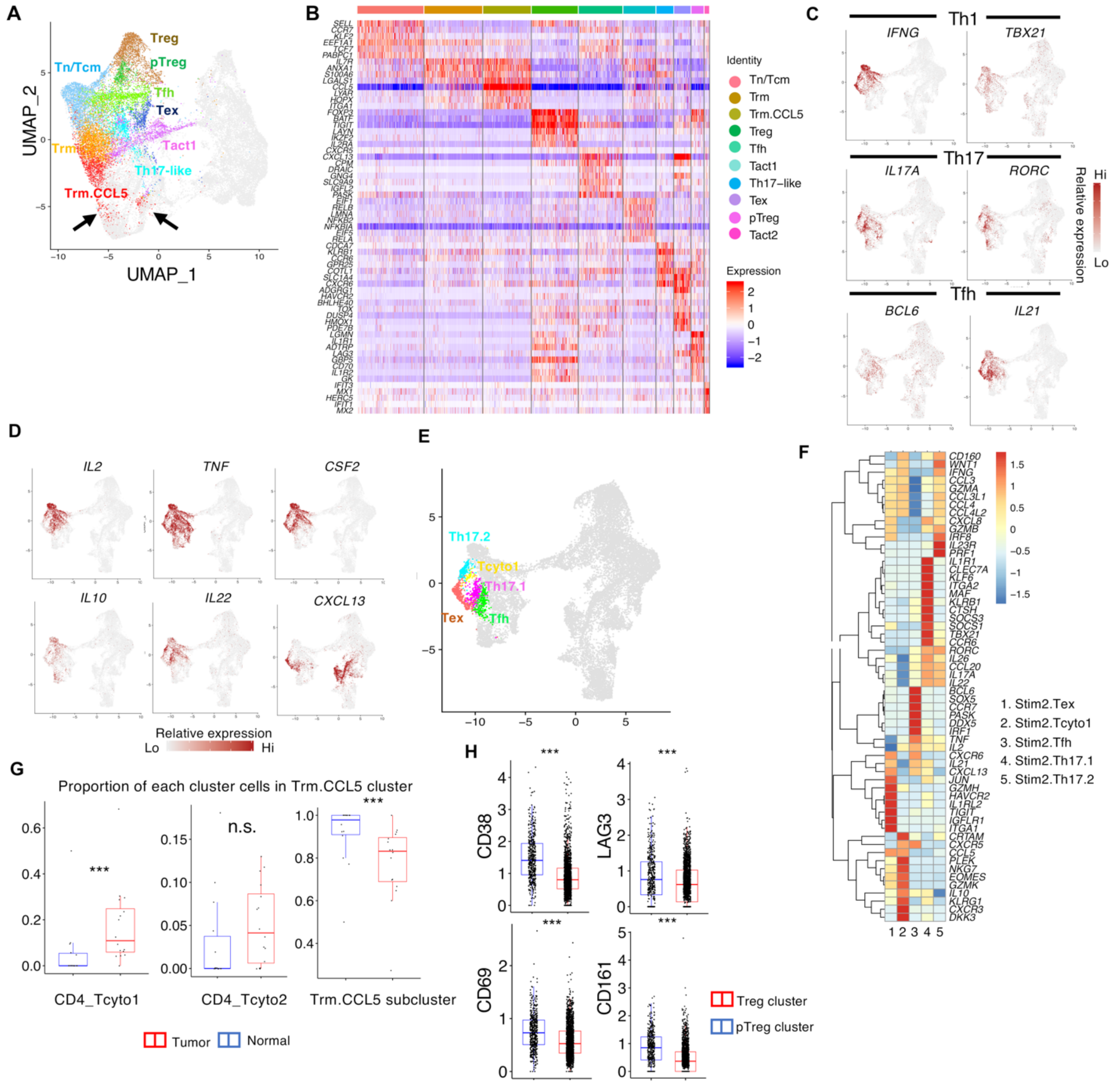

Supplemental Figure 5. Characterization of effector CD4$^+$ T cells within tumors. (**A**) Distribution map of non-stimulated CD4$^+$ T cells (in Figure 4A) on the total T cell UMAP (in Figure 1B). Arrows indicate CD4$^+$ T cells within the T-Tcyto1 and T-Tcyto2 clusters. (**B**) Heatmap of differentially expressed genes between cells in each CD4 non-stimulated cluster. (**C**) Heatmap of marker genes for Th1, Th17, or Tfh cells on the CD4 UMAP. (**D**) Heatmap of cytokine and effector genes (as indicated) on the CD4 UMAP. (**E**) Distribution of cells within each CD4_Stim2 subcluster on the CD4 UMAP. (**F**) Heatmap of differentially expressed genes (as indicated) for cells within each CD4_Stim2 subcluster. (**G**) Proportion of normal cells or intratumoral cells within each subcluster of CD4_Trm.CCL5 cluster for each patient (n=16) and its comparison. *** *P* (Wilcoxon test) <0.01; * *P* <0.05; n.s.(not significant) (**H**) Comparison of ADT signal (as indicated) between CD4_pTreg cluster and CD4_Treg cluster cells. *** *P* (Wilcoxon test) <0.0001

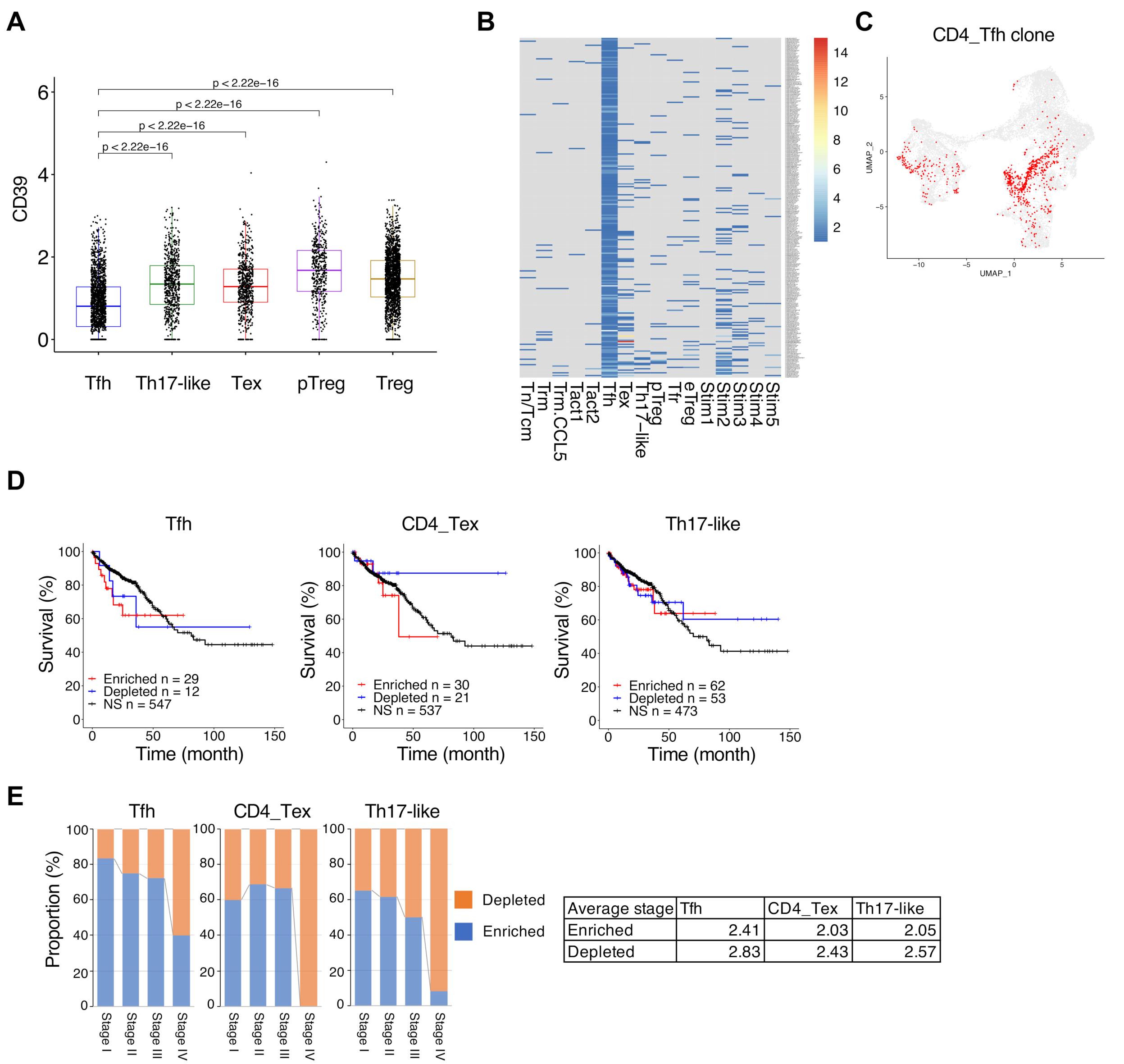

Average stage	Tfh	CD4_Tex	Th17-like
Enriched	2.41	2.03	2.05
Depleted	2.83	2.43	2.57

Supplemental Figure 6. Lineage and prognostic significance of peripheral CD4$^+$ T cells in CRC. (**A**) Comparison of CD39 expression in between CD4_Tfh and other effector clusters as indicated. *P*-values (Wilcoxon test) were indicated. (**B**) Heatmap of Tfh T cell clonotypes among the CD4 non-stimulated and stimulated clusters. (**C**) All expanded T cell clones from cluster CD4_Tfh on the CD4 UMAP. (**D**) Kaplan–Meier curves of overall survival in the CRC TCGA cohort for patients enriched or depleted for CD4_Tfh, CD4_Tex, or CD4_Th17-like gene sets by GSEA (Methods). (**E**) Bar graphs depicting relative proportion of patients by stage enriched (blue) or depleted (orange) for the gene sets. Average stage is indicated on right.

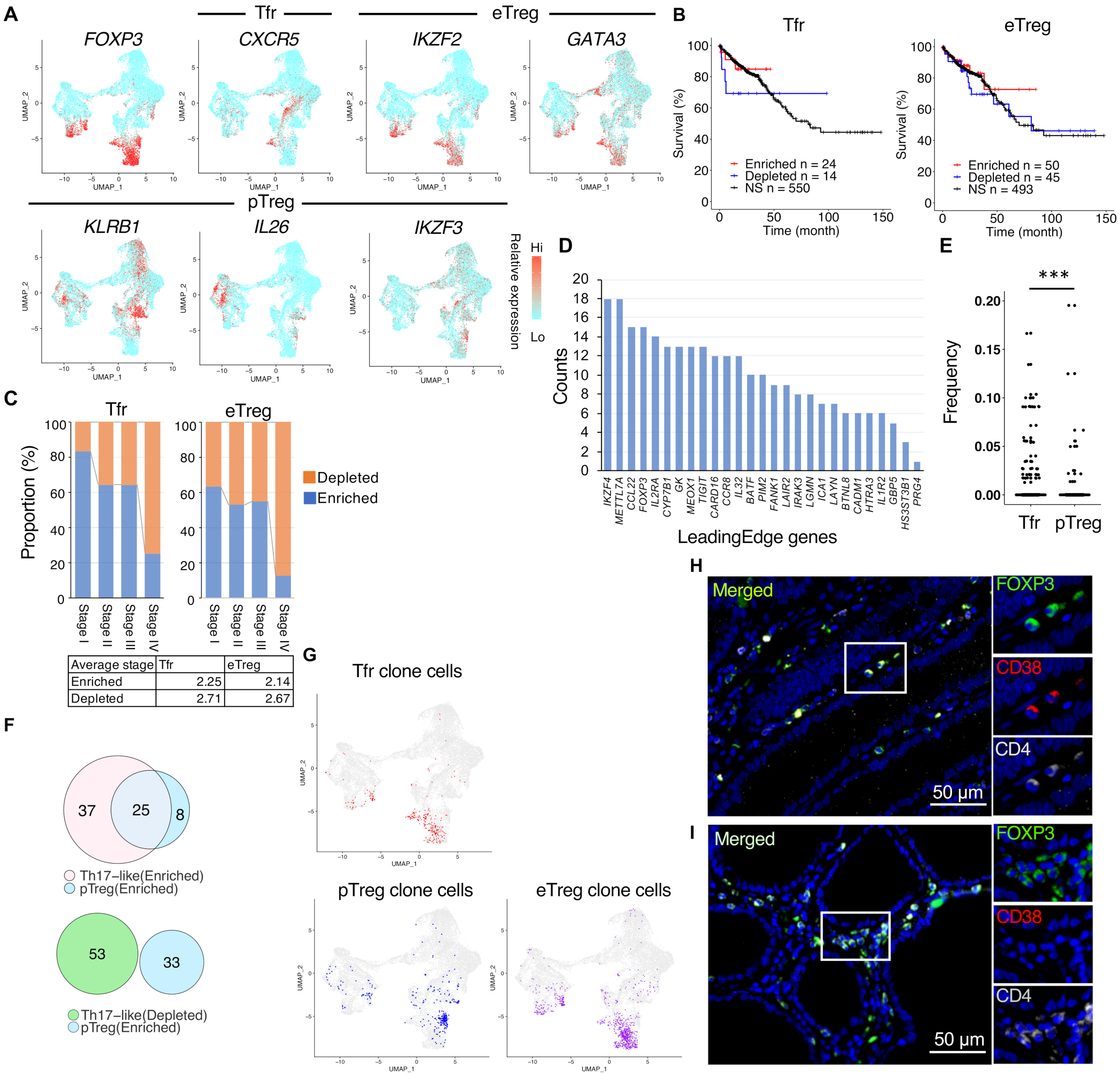

Supplemental Figure 7. Characterization of Treg subpopulations in CRC. (**A**) Relative gene expression of marker genes (as indicated) for pTreg, eTreg, or Tfr cells on the CD4 UMAP (in Figure 4A). (**B**) Kaplan-Meier curves of overall survival in the CRC TCGA cohort for patients enriched or depleted for CD4_Tfr or CD4_eTreg gene sets by GSEA (Methods). (**C**) Bar graph depicting relative proportion of patients by stage enriched (blue) or depleted (orange) for Tfr or eTreg subclusters. Average stage is indicated (bottom). (**D**) Leading-edge genes of the patients enriched for the positively prognostic T-Treg cluster in Figure 2C. (**E**) eTreg TCR clonotype frequencies shared with Tfr or pTreg; *** $P < 0.0001$ (Wilcoxon test). (**F**) Venn diagram showing the number of patients enriched for Th17 and pTreg genes (top). The same is shown for the number of patients depleted for Th17 and enriched for pTreg (bottom). (**G**) All expanded T cell clones present in Tfr (left), eTreg (right) or pTreg (bottom) subsets on the CD4 UMAP. (**H** and **I**) IHC staining with anti-human CD4, -CD38, and -FOXP3 antibodies respectively to validate target T cells, $CD38^{+}$ Tregs (pTregs) (**H**) or $CD38^{-}$ Tregs (**I**) in stage 2/3 CRC (n = 2, exemplified by the patient A076 or A004, respectively, see Supplemental Table 10).

Supplemental Materials

Materials	Source	Cat. Number
Percoll	Millipore-Sigma	Cat# GE17-0891-01
Dynabeads™ MyOne™ Silane	Thermo	Cat# 37002D
SPRIselect	Beckman Coulter	Cat# B23318
TCO-PEG4-NHS Ester	Click Chemistry	Cat# A137-10
Methyltetrazine-PEG4-NHS Ester	Click Chemistry	Cat# 1069-10
20X Borate Buffered Saline	Thermo	Cat# 28341
UltraPure Glycine	Thermo	Cat# 15527013
Chromium Single Cell 5' Library Construction Kit, 16 rxns	10x Genomics	Cat# 1000020
Chromium Single Cell A Chip Kit	10x Genomics	Cat# 120236
Kapa HiFi HotStart PCR ReadyMix	Kapa Biosystems	Cat # KK2601
Tumor Dissociation Kit, human	Miltenyi	Cat # 130-095-929
SI-PCR Primer	10x Genomics	N/A
Chromium i7 Sample Index	10x Genomics	https://support.10xgenomics.com/single-cell-gene-expression/index/doc/specifications-sample-index-sets-for-single-cell-3

HTO Cell Hashing cDNA additive primer: GTGACTGGAGTTCAGACG TGTGCTC	NYGCtech	https://cite-seq.com/protocol/
ADT CITE-seq cDNA additive primer: CCTTGGCACCCGAGAATTC C	NYGCtech	https://cite-seq.com/protocol/
Illumina i7 index1	Illumina	https://support.illumina.com/downloads/illumina-adapter-sequences-document-1000000002694.html

Supplemental Table 1. Patient nomenclature

Patient Normal (N)/Tumor (T)	Date of surgery	Patint ID	Stage	Pre-treatment	sex	age
pt01 N	11/15/17	TC-040	IIA	No	male	66
pt01 T	11/15/17	TC-040	IIA	No	male	66
pt02 N (MSI-H)	2/28/17	TC-001	IIA	No	male	32
pt02 T (MSI-H)	2/28/17	TC-001	IIA	No	male	32
pt03 N	3/29/17	TC-002	I	No	male	70
pt03 T	3/29/17	TC-002	I	No	male	70
pt04 N	5/3/17	TC-005	IIA	No	male	67
pt04 T	5/3/17	TC-005	IIA	No	male	67
pt05 N	5/18/17	TC-007	I	No	male	71
pt05 T	5/18/17	TC-007	I	No	male	71
pt06 N	6/15/17	TC-009	IIA	No	male	71
pt06 T	6/15/17	TC-009	IIA	No	male	71
pt07 N	11/22/17	TC-041	IIIA	No	female	65
pt07 T	11/22/17	TC-041	IIIA	No	female	65
pt08 N	6/28/17	TC-012	I	No	female	62
pt08 T	6/28/17	TC-012	I	No	female	62
pt09 N	9/27/17	TC-033	IIIA	No	male	46
pt09 T	9/27/17	TC-033	IIIA	No	male	46
pt10 N (MSI-H)	12/13/17	TC-048	IIIA	No	female	88
pt10 T (MSI-H)	12/13/17	TC-048	IIIA	No	female	88
pt11 N	4/11/19	TC-064	IIA	No	male	58
pt11 T	4/11/19	TC-064	IIA	No	male	58
pt12 N	6/29/17	TC-013	IIIC	No	male	60
pt12 T	6/29/17	TC-013	IIIC	No	male	60
pt13 T	7/20/17	TC-019	I	No	female	69
pt14 N	7/26/17	TC-021	IIA	No	male	69
pt14 T	7/26/17	TC-021	IIA	No	male	69
pt15 N	12/6/17	TC-043	I	No	male	84
pt15 T	12/6/17	TC-043	I	No	male	84
pt16 N	12/13/17	TC-046	IIC	No	male	60
pt16 T	12/13/17	TC-046	IIC	No	male	60

Supplemental Table 2. Antibody clones and barcodes for CITE-seq and Cell Hashing

Antibody	Clone	CITE-seq Barcode
CD27	M-T271	TAGAACCAACAC
CD103	Ber-ACT8	TAGACTGGCAAC
PD-1	NAT105	TAGCAACTGCTC
HLA-DR	L243	TAGCGTGTTCTG
CD39	A1	TAGGAGAGTTGT
CD25	M-A251	TAGGTGGATCCT
CD57	HNK-1	TAGTACCCTGGT
CD137	4B4-1	GATAAGCAGGTA
CD45RA	HI100	GATAGCGCTAAG
CD161	HP-3G10	GATCATGGATAG
CD69	FN50	GATCGACCTTCA
CD45RO	UCHL1	GATGATCCCTGA
LAG3	11C3C65	GATGTCATCACT
TIGIT	A15153G	GATTCATCGCTG
CD4	OKT4	CTACTTGGTACA
CD8	HIT8a	CTAGAACAGAGG
CTLA4	BNI3	CTAAAGTCCCAA
CCR7	G043H7	CTAAGACGCAGA
CD44	BJ18	CTAATTACTCGG
CD38	HIT2	ATCACAAGTGTC
TIM3	F38-2E2	ATCAGTCACTAG
CD62L	DREG-56	ATCCCAATCATG
KLRG1	SA231A2	ATCCGTTCGAAA
ICOS	C398.4A	ATCGCTCCTATG
OX40	BER-ACT35	ATCGTGAGAGCA

Supplemental Table 3. Experimental datasets for scRNA-seq, TCR-seq, and CITE-seq

Patient/Date	Patient ID	May 29 exp. * (5/29/19)	June 16 exp. * (6/16/19)	June 23 exp. *, # (6/23/19)	Aug 13 exp.*, ## (8/13/19)
2/28/17	TC-001		Hash54 T, Hash56 N		Hash51 T
3/29/17	TC-002		Hash1 T, Hash2 N		Hash5 T, Hash6 N
5/3/17	TC-005		Hash57 T, Hash58 N		
5/18/17	TC-007		Hash51 T, Hash52 N		Hash1 N
6/15/17	TC-009		Hash3 T, Hash4 N		
6/28/17	TC-012			Hash59 T, Hash60 N	
6/29/17	TC-013				Hash2 T, Hash4 N
7/20/17	TC-019				Hash54 T
7/26/17	TC-021				Hash57 T, Hash56 N
9/27/17	TC-033			Hash54 T, Hash56N	
11/15/18	TC-040	Hash3 T, Hash4 N		Hash57 T, Hash58 N	
11/22/18	TC-041			Hash51 T, Hash52N	
12/6/18	TC-043				Hash3 T, Hash58 N
12/13/17	TC-046				Hash59 T, Hash60 N
12/13/17	TC-048			Hash3 T, Hash4 N	
4/11/19	TC-064			Hash1 T, Hash2 N	

*Stimulated/non-stimulated: Stimulated Hash11, Non-stimulated Hash9
Sample 1: Hash51, Sample2-1 and Sample2-2: All except Hash51
Sample1: Hash 1, 2, 3, 4, 5, 6, 51, 54, 56, 57, 58, 59, 60, Sample2: Hash 1, 4, 6, 56, 58, 60

Supplemental Table 4. Cluster cell number by patient and sample

	Number of cells(Tumor)	Number of cells(Normal)	Number of cells(Total)
T-Trm	5399	2335	7734
T-Tn/Tcm	3780	443	4223
T-Treg	2855	200	3055
T-Tex	2242	156	2398
T-Tcyto1	1916	250	2166
T-Tcyto2	1564	378	1942
T-Tact	966	373	1339
T-Stim1	5242	1866	7108
T-Stim2	4551	629	5180

Patient Tumor	T-Trm	T-Tn/Tcm	T-Treg	T-Tex	T-Tcyto1	T-Tcyto2	T-Tact	T-Stim1	T-Stim2	Color (patient)
pt01 T	967	975	543	574	284	227	95	475	436	yellow
pt02 T	85	43	146	89	21	41	115	157	221	limegreen
pt03 T	157	135	133	73	87	48	14	172	198	gold
pt04 T	65	351	48	34	24	19	5	46	166	blue
pt05 T	142	199	170	46	24	14	91	54	211	cyan
pt06 T	892	390	272	100	305	176	192	453	503	darkorchid4
pt07 T	606	413	485	564	148	239	59	572	931	pink
pt08 T	449	344	142	88	135	43	131	392	203	green
pt09 T	70	63	42	19	29	11	10	49	27	firebrick1
pt10 T	649	286	152	155	275	264	72	728	190	navy
pt11 T	76	34	8	11	26	4	14	87	11	darkolivegreen1
pt12 T	234	46	33	7	44	28	34	178	94	plum2
pt13 T	532	163	243	215	254	126	71	884	520	sandybrown
pt14 T	83	18	99	114	51	141	27	215	68	mediumpurple1
pt15 T	218	122	113	65	49	44	23	574	282	coral
pt16 T	174	198	226	88	160	139	13	206	490	cadetblue1

Patient Normal	T-Trm	T-Tn/Tcm	T-Treg	T-Tex	T-Tcyto1	T-Tcyto2	T-Tact	T-Stim1	T-Stim2	Color (patient)
pt01 N	371	93	109	105	42	47	17	190	94	cadetblue1
pt02 N	36	21	4	4	10	6	17	24	36	coral
pt03 N	11	5	6	1	9	6	1	21	5	mediumpurple1
pt04 N	38	31	4	4	1	NA	5	8	30	sandybrown
pt05 N	13	3	2	NA	NA	3	7	19	7	plum2
pt06 N	1278	182	58	25	125	230	242	570	267	mediumpurple
pt07 N	44	9	NA	1	2	11	2	3	NA	firebrick1
pt08 N	129	31	1	2	4	16	12	116	15	green
pt09 N	51	8	NA	1	7	1	5	67	14	pink
pt10 N	65	4	4	7	5	7	3	200	42	aquamarine
pt11 N	80	8	1	1	2	5	5	457	6	cyan
pt12 N	45	9	NA	1	26	5	4	43	9	blue
pt14 N	134	33	11	4	8	8	51	71	89	gold
pt15 N	2	2	NA	NA	1	2	NA	20	2	limegreen
pt16 N	38	4	NA	NA	8	31	2	57	13	magenta

Supplemental Table 5. Gene sets for GSEA (related to Figure 2 and Supplemental Figure 3)

T-Treg	T-Tex	T-Tcyto1	T-Tcyto2
FOXP3	CXCL13	GZMK	GNLY
BATF	GNG4	FGFBP2	CCL5
TBC1D4	CPM	EOMES	KLRD1
TIGIT	PDE7B	NKG7	CD8A
ICA1	NUSAP1	SAMD3	NKG7
GK	AHI1	DKK3	KLRC1
ADTRP	TSPAN13	CST7	CD8B
RTKN2	MS4A6A	CD8A	PRF1
LGMN	IGFL2	PACSIN1	SERINC2
STAM	TTC21A	PPP1R14B	FXYD2
PRG4	PDLIM4	C1orf21	CTSW
HTRA3	SLC1A4	CMC1	AOAH
LAIR2	G0S2	CXCR3	SLC27A2
GBP5	ASCL2	CD8B	CAPG
GNG8	TOX	MS4A1	KLRC3
IL1R2	MYO7A	S1PR5	C12orf75
CYP7B1	CAMK1	HLA-DOA	C9orf139
METTL7A	BUB1B	GZMA	FCRLB
PIM2	SPRED3	PLXDC1	DBN1
CARD16	BCAT1	PDLIM1	CD244
ZC2HC1A	EVC2	LGR6	SLC17A9
RASGRP4	LRRC8D	KLRG1	PIK3AP1
TNFRSF1B	KSR2	ITM2C	RAB13
MEOX1	HMOX1	CX3CR1	CD101
IRAK3	PTPN14	VIPR2	GZMH
HS3ST3B1	IGHM	PECAM1	TMCC3
CCR8	BARX2	RCAN2	PLEKHA4
IL32	LMCD1	ENC1	GZMB
LAYN	NR3C1	PRR5L	PRR5L
FANK1	COTL1	CD300A	YPEL1
UTS2	DRAIC	CHI3L2	CD63
TNFRSF18	MYL6B	SMARCD3	TSHZ3
IKZF4	TEX48		GALNT2
BTNL8	PHEX		IMPA2
CADM1			NCR1
SKAP1			GZMA

SIRPG			SH3BGRL3
CCL22			NUDT13
IL2RA			AVEN

CD4_Tfh	CD4_Th17-like	CD4_Tex	CD4_pTreg	CD4_Tfr	CD4_eTreg
CD4	CD4	CD4	CD4	CD4	CD4
CXCR5	TMIGD2	CXCL13	LGMN	FOXP3	FOXP3
CPM	CXCR6	ADGRG1	FOXP3	BATF	RTKN2
CXCL13	SLC1A4	TSPAN13	ADTRP	DUSP4	IL32
DRAIC	CDCA7	GNG4	ZC2HC1A	CSNK1G3	METTL7A
SLC9A9	JAML	ADAMTS6	IL1R1	STAM	LAIR2
ANKRD55	KLRB1	RNF215	GBP5	FCRL3	LAYN
IGFL2	EGLN3	SLC1A4	ZZEF1	CCR8	CD177
PASK	GPR25	HMOX1	GPR25	GRSF1	CCL22
GNG4	COTL1	AHI1	RASGRP4	PMAIP1	BATF
NEK6	FSD1	G0S2	LAG3	LAYN	CARD16
TLR5	ITGAE	ETV7	CACYBP	CHST7	TIGIT
NR3C1	MSC	ZBED2	IL1R2	TNFRSF18	IKZF2
CHGB	ASB2	PRR5L	HSDL2	IKZF4	STAM
ARMH1	GFPT2	PDE7B	CD70	FAS	TBC1D8
FKBP5	ALOX5AP	HIST1H2BH	UTS2	GADD45A	TBC1D4
CABLES2	GPR55	ADGRG5	HS3ST3B1	CCL22	TTN
IGFBP4	BRIP1	U91328.1	TNFRSF1B	SEC11C	ICA1
EFCAB13	OSTF1	DUSP4	CMTM6	GOT2	CD74
MICALL2	SMCO4	MYO1E	SKAP1	EBI3	FANK1
LBHD1	PLEKHF1	PDLIM4	SMCO4	CHRNA6	CDCA2
PPFIBP2	ABI3	FMO5	FAM174B	CD80	ZC2HC1A
BFSP2	IRAK1BP1	MYO7A	UCP2	GK	IKZF4
VASH1	MYO1F	NR5A2	ZNRF1	CEP120	ACP5
HIST1H4I	CA2	ADAM28	MEOX1	TIGIT	IRAK3
TSHZ2	MGLL	LIMS2	TRIB2	CADM1	MEOX1
TIGIT	CD3G	ZNF367	CACNA2D2	NOP58	CD27
HCG25	AURKA	ENTPD1	CD302	PIM2	MAGEH1
TMEM123	GLDC	FSIP1	JCHAIN	C16orf87	GLRX
RAB3D	OTUB2	OTUB2	DNPH1	UBC	PMCH
ACTN1	ADAM12	LMCD1	C20orf96	BCL2L1	PIP5K1B
IL6R	SH3BGRL3	HAVCR2	DBF4B	MOB1B	ZG16B
MEOX1	SOX5	PDGFA	OTUB2	CARD16	CABLES1
PDE7B	DAPK2	PM20D2	MACC1	MIIP	PIM2

SULT1A1	CENPE	UPK1A	MT1E	TSPAN5	HS3ST3B1
SMCO4	S100A4	TMEM244	BLM	ZC3H7A	ADTRP
LEF1	BLK	TRGC1	CTSH	LY75	HLA-DMA
SESN3	PLD1	BTBD11	SAMHD1	ATOX1	CADM1
LAT	MACC1	AURKA	UAP1L1	RAB9A	SKAP1
INAFM2	ZNF81	BFSP2	VCPIP1	TBC1D4	ARHGEF5
DCBLD1	CABLES2	L1CAM	ACP5	SNHG15	HSPA1A
TCF7	IGHA1	HIST1H4F	ASB2	ICA1	CARD17
NMB	MGAT4A	CXCR6	CHST2	SMG9	HLA-DRB1
C5orf17	SMIM3	CPM	SALL2	ZNF821	TNFRSF1B
CLHC1	TNFSF13B	SLC2A8	CDKL2	HTATIP2	TMSB10
USP31	PARPBP	CCDC136	CD247	RNF114	CYP7B1
FGFR1	CBX8	ABCA2	TPRN	SNX11	UTS2
CLBA1	GZMA	DIRC2	RNF213	KCNN4	MT1E
PTPN13	MYO7A	TSPAN2	P2RY14	BCL2	GNG8
ASB13	CEBPD	HOMER2	STRIP2	NFKB2	SLC4A5
MORN4	FAM167A	NOS3	TAPT1-AS1	SFT2D1	CCR8
MAFTRR	SLC25A35	PLXNB2	CYTH1	NCF2	HLA-DPB1
ITGB8	SYTL2	C21orf58	KANSL1L	CACYBP	ENTPD1
LRRC8D	LIMS1	RFX2	HIST1H2BH	CASZ1	SMAD1
ST6GALNAC1	OGFRL1	ADAMTS10	TBC1D31	RGS1	EEPD1
ITM2A	YPEL2	RAD52	ZBTB38	TBC1D8	SLC12A5
C8orf37	TRPV1	EXT1	IL6R	TNIP3	CSF2RB
CXCR4	SLC4A7	HMSD	ABI3	TANK	CLNK
IL6ST	ENTPD1	SLC1A1	FURIN	TFRC	CNKSR2
IGHM	CD3D	IQCC	KCNAB3	C6orf48	PIK3R3
TCTN2	ADAM28	FUCA1	ZNF282	ETV7	SELL
MORN1	GRAP2	FANCI	FTL	CCDC50	HLA-DPA1
FAM216A	THNSL1	SNTB1	FAM215B	STAC	LIPC
AFMID	HDLBP	TPK1	HOXB3	ANKRD10	ZNF80
ZNF343	RAP1B	NUSAP1	LRP2BP	OAZ1	PNMA5
MGAT5B	B3GALT2	AFAP1L2	RABGAP1L	DNAJA1	B2M
ZSCAN20	ELOVL6	JAML	IKZF3	HERPUD1	SAMHD1
CHN1	LSP1	TOX	ZNF425	IL21R	GRK3
KRTCAP3	KDM1B	PIP5K1B	AKIP1	CRADD	DNPH1
CHI3L2	TBXAS1	JRK	IL2RB	TRAF3	HDAC9
NFIA	CD96	S100P		YAF2	RASGRP4
RAB37	LYN	BUB1B		JAKMIP1	HPGD
TOX	KIT	SOX4		ZNF292	CMTM7

TRDV1	ZNF519	MAFTRR		TNFRSF8	JAKMIP1
PHETA2	ODAPH	PON2		DEPDC7	C15orf53
LRG1	CCNJ	CTSL			CARD9
NSG1	CD52	RAD54B			ACOXL
ALG1L	HMGB1	PRRG4			CD302
TMCC2	THEMIS	ATP8B4			PTGIR
SESN1	GYG1	DBN1			AKAP2
ULK2	H2AFY2	KLHL3			HLA-A
ITGA4	RNF215	IGFLR1			AVPI1
FYB1	MVB12B	POU2AF1			VAV3
SPATA1	CD2	MCM5			FNIP2
FAM153B	ADA	CAMK1			SOX4
FAM19A2	NUDT17	ZWINT			CPNE2
KIAA1324	GAS2L1	SLX4			HLA-C
GAREM2	ACP5	TMEM164			ZNF821
RAD52	CCDC146	HSPA1L			UCP2
MYL6B	CHAF1B	MCAM			HMOX1
COTL1	MAP3K4	E2F1			EPHX2
AFAP1L2	ATP10D	TVP23C			COCH
SCML1	FKBP11	WWTR1			OAS1
TOP2A	PRC1	FBXL22			
ZNF823	GAB3	LONRF3			
RAB40C	TROAP	NDC80			
PCBP3	LCK	LHFPL6			
TRIM46	LAIR1	COTL1			
MAP3K7CL	LBX2	KIF18A			
AKAP2	S100A6				

Supplemental Table 6. P-values for Kaplan Meier analysis (related to Figure 2 and 5 and Supplemental Figure 3, 6, and 7)

	Enriched vs NS	Depleted vs NS	Enriched vs Depleted
T-Tcyto1	0.45	6.90E-03	0.029
T-Tcyto1 (Stage II-III)	0.2	3.90E-02	0.023
T-Tcyto2	0.38	0.49	0.2
T-Tcyto2 (Stage II-III)	0.5	0.82	0.87
T-Treg	0.38	0.021	0.019
T-Treg (Stage II-III)	0.28	0.056	0.062
T-Tex	0.4	0.18	0.1
T-Tex (Stage II-III)	0.45	0.51	0.33
CD4_pTreg (CRC)	0.023	0.29	0.035
CD4_pTreg (CRC) StageII-III	0.013	0.16	0.0065

	Enriched vs NS	Depleted vs NS	Enriched vs Depleted
T-Tcyto1(melanoma)	2.80E-03	0.97	0.035
T-Tcyto2(melanoma)	0.012	0.68	0.014
T-Treg(melanoma)	0.05	0.34	0.57
CD4_pTreg (BRC)	0.13	0.094	0.019
CD4_Tfr	0.85	0.45	0.24
CD4_eTreg	0.52	0.7	0.33
CD4_Tfh	0.079	0.48	0.78
CD4_Tex	0.61	0.29	0.35
CD4_Th17-like	0.5	0.91	0.98

Supplemental Table 7. MAIT cell distribution in the CD8 UMAP

	MAIT(tumor)	MAIT(Normal)
Total cell number	199	57
pt01	17	6
pt02	3	0
pt03	9	0
pt04	3	0
pt05	6	0
pt06	11	2
pt07	15	0
pt08	10	2
pt09	7	2
pt10	6	1
pt11	5	41
pt12	15	0
pt13	15	0
pt14	5	2
pt15	64	1
pt16	8	0

Clusters	MAIT(Tumors)	All(Tumors)	(MAIT vs All) %	MAIT(Normal)	All (Normal)	(MAIT vs All) %
Tn/Tcm	0	261	0.00	1	33	3.03
Trm	51	1693	3.01	7	628	1.11
Tem	9	915	0.98	2	159	1.26
Temra	2	221	0.90	1	18	5.56
IEL	12	540	2.22	15	445	3.37
Tex	34	1925	1.77	2	85	2.35
Tact	7	459	1.53	0	161	0.00
Stim1.1.Trm	9	290	3.10	0	187	0.00
Stim1.2.Trm	7	925	0.76	11	551	2.00
Stim2.1.Tem	18	576	3.13	1	92	1.09
Stim2.2.Tem	1	135	0.74	0	8	0.00
Stim3.IEL	8	508	1.57	13	387	3.36
Stim4.Tex	41	1194	3.43	4	66	6.06

Supplemental Table 8. Gene lists highly correlated with marker genes *TCF7*, *FGFBP2*, or *HAVCR2*

Correlated gens	R-value	Correlated gens	R-value	Correlated gens	R-value
TCF7	1	HAVCR2	1	FGFBP2	1
CCR7	0.314	HMOX1	0.36	FCGR3A	0.493
SELL	0.247	PRF1	0.346	GZMH	0.38
IL7R	0.24	LAG3	0.34	C1orf21	0.378
S1PR1	0.236	GNLY	0.335	PRSS23	0.373
PASK	0.234	GZMA	0.321	LITAF	0.326
RPL13	0.23	CCL5	0.3	ZNF683	0.319
RPS12	0.226	LRRC28	0.29	CST7	0.277
LEF1	0.221	CD27	0.288	APOBEC3G	0.242
ICAM2	0.221	GALNT2	0.277	OASL	0.24
RPS8	0.22	CTSW	0.275	ASCL2	0.226
RPL39	0.218	NKG7	0.273	PLEK	0.218
TPT1	0.212	PTMS	0.253	KLRG1	0.214
EEF1A1	0.211	ACP5	0.251	CD52	0.212
PABPC1	0.203	CXCR6	0.243	C12orf75	0.211
RPS18	0.203	ADGRG1	0.239	SAMD3	0.208
RPL34	0.199	GZMB	0.235	CARMIL3	0.195
RPS2	0.197	C12orf75	0.234	CX3CR1	0.194
CXCR4	0.196	LSP1	0.231	HLA.B	0.193
RPS3A	0.195	CSF1	0.228	CXCR3	0.192
RPL9	0.192	MCM5	0.225	RASA3	0.189
LTB	0.186	CXCL13	0.221	YWHAQ	0.186
RPS14	0.184	LYST	0.218	GNLY	0.185
RPL3	0.183	TMSB10	0.212	S1PR5	0.183
RPS16	0.18	CD74	0.21	TSPAN2	0.182
RPS21	0.178	IGFLR1	0.206	AC010616.1	0.182
TAGLN2	0.173	IL32	0.203	S1PR1	0.181
N4BP3	0.173	ITGB2	0.203	PRR5L	0.181
SPINT2	0.172	CD3D	0.203	HMOX1	0.18
KLF2	0.17	VCAM1	0.202	TMSB10	0.179
		NDFIP2	0.198	SELPLG	0.175
		UCP2	0.197	GTSF1	0.173
		HCST	0.194	GZMA	0.169
		ENTPD1	0.192	TENT5A	0.169

		CD63	0.187	TGFBR3	0.166
		LITAF	0.18	HLA.A	0.162
		CLEC2D	0.179	NOD2	0.162
		HLA.DRB1	0.178	GZMK	0.161
		SLF1	0.178	P2RY8	0.161
		LINC01943	0.173	ITGB1	0.16
		TNFSF4	0.172	APMAP	0.159
		HOPX	0.171	ITGB2	0.158
		SNAP47	0.171	HDAC4	0.158
		CD82	0.17	SH3BP5	0.156
		BATF	0.169	FLNA	0.155
		CTSC	0.167		
		RIN3	0.166		
		DUSP4	0.165		
		BHLHE40	0.165		
		LY6E	0.165		
		AC243829.4	0.165		
		PDCD1	0.164		
		AD000671.2	0.163		
		CORO1A	0.162		
		DGKZ	0.162		
		HLA.DPA1	0.161		
		TRAF3IP3	0.161		
		SIT1	0.161		
		HLA.DMA	0.161		
		APOBEC3C	0.16		
		SOX4	0.159		
		ANXA5	0.158		
		LINC01871	0.157		
		SYNGR2	0.157		
		ITGB1	0.156		
		IFI27L2	0.156		
		TSPO	0.155		
		CST7	0.153		
		ITGAL	0.153		
		AFAP1L2	0.153		
		GZMH	0.151		
		TSPAN13	0.15		
		RDH10	0.15		

		APOBEC3G	0.149		
		LAYN	0.147		
		IL2RB	0.147		
		CBLB	0.146		
		TNFRSF9	0.145		
		CARD16	0.145		
		PYCARD	0.144		
		MCTP2	0.144		
		MYO1G	0.143		
		B2M	0.141		
		HLA.DRA	0.14		
		TIGIT	0.14		
		FGL2	0.14		
		IFITM10	0.14		
		SIRPG	0.14		
		TMC8	0.138		
		GBP5	0.137		
		BST2	0.136		
		ITGA2	0.135		
		EVL	0.134		
		FXYD2	0.133		
		TNFRSF1B	0.133		
		CCR1	0.133		
		LIMD2	0.133		
		CD109	0.132		
		OAS1	0.131		
		FUT8	0.131		
		PDE4DIP	0.13		
		INPP5F	0.13		
		MPST	0.129		
		LGALS9	0.128		
		HLA.DQA1	0.127		
		EID1	0.126		
		S100A4	0.125		
		TNIP3	0.125		
		MYL12A	0.125		
		SNX9	0.124		
		CCND2	0.123		
		ALOX5AP	0.122		

		KLRD1	0.122		
		LAIR2	0.122		
		PGAM1	0.122		
		ARL4C	0.121		
		CD2	0.121		
		SLC9A3R1	0.121		
		TTC24	0.121		
		SLC2A8	0.119		
		BCL2L11	0.119		
		SH3BP1	0.119		
		PLS3	0.118		
		HLA.DRB5	0.118		
		PRDM1	0.118		
		PTPN7	0.118		
		NAMPT	0.117		
		IFITM2	0.117		
		ID2	0.116		
		SMC4	0.116		
		NFATC3	0.116		
		DNPH1	0.115		
		RBPJ	0.115		
		ANXA11	0.114		
		PPM1M	0.114		
		PCED1B	0.113		
		NPDC1	0.113		
		ARPC1B	0.113		
		SLC4A7	0.113		
		PHLDA1	0.112		
		KIAA0825	0.112		
		RAC2	0.112		
		IFI6	0.111		
		AC004865.2	0.111		
		NR5A2	0.111		
		ATP10D	0.111		
		BLOC1S1	0.111		
		NEAT1	0.11		
		TOX	0.11		
		ADGRG5	0.11		
		JAKMIP1	0.11		

		PSMB8	0.11		
		CHST12	0.109		
		WHRN	0.109		
		VAMP5	0.109		
		ARL3	0.109		
		CTLA4	0.108		
		MIR155HG	0.108		
		CD70	0.108		
		MTSS1	0.108		
		LINC01358	0.108		
		SPTAN1	0.107		
		TRGV2	0.106		
		HLA.DPB1	0.106		
		HDLBP	0.106		
		LBH	0.106		
		CAMK1	0.106		
		ZBED2	0.105		
		SH2D1A	0.104		
		SLC27A2	0.104		
		PLA2G16	0.104		
		PAG1	0.103		
		TP73	0.103		
		CCL4	0.102		
		SETBP1	0.102		
		FASLG	0.101		
		RARRES3	0.101		
		NAP1L4	0.101		
		CTNNB1	0.101		
		RAC1	0.101		
		FGFBP2	0.1		
		NELL2	0.1		
		HMGN3	0.1		
		GPD2	0.1		

Supplemental Table 9. Gene lists for cell trajectory analysis (related to Figure 3I and Supplemental Figure 4F)

CD8_Tn/Tcm	CD8_Trm	CD8_Tem	CD8_Temra	CD8_IEL	CD8_Tex
SELL	RPLP1	GZMK	FGFBP2	KIR3DL1	CXCL13
S1PR1	AQP3	ADCK1	ZNF683	KIR3DL2	B2M
KLF2	RPLP0	DKK3	GZMH	LAT2	CXCR6
FBLN7	USP13	SMARCD3	FCGR3A	GNLY	TMSB10
ANK1	TRIQK	OTUD1	CLEC11A	TRGV4	LSP1
TIMP1	GPR15	CATSPER2	NOD2	FAM49A	HLA-DPA1
SPTY2D1OS	FBLN5	NOD2	GNLY	ZNF683	COTL1
IL7R	RPS12	LGALS3BP	CARMIL3	CD52	HLA-DRA
PASK	IL7R	IGFBP4	MROH6	ZNF891	GPR25
SLC9A7	ZNF629	C3orf62	NKG7	CCDC102B	IL32
LEF1	NCF1	C1orf21	TM6SF1	IFI30	ITGB2
CCR7	RHEBL1	ARRDC5	ROBO3	ZNF595	SOX4
RAB11FIP5	FTL	ATP9A	DLG4	HOPX	LCK
TNFSF13B	CKAP4	ZBTB39	GSAP	B4GAT1	CD74
TCF7	DNHD1	CXCR3	TTYH2	LYPD3	HLA-DRB5
CRYBG3	TEC	CRYBB2	HDAC4	MIS18A	CORO1A
ACCS	S100A6	HIRA	MATK	CCL5	CD3D
PDE9A	NUAK2	RPL28	KCNK12	TAGLN2	PLEKHF1
RHEBL1	TNFSF13B	RPS27	C1orf21	RPLP1	CTSW
RASGRP2	ESPN	RPS14	ARL4D	RARRES3	LAPTM5
GLCE	TMEM143	CARMIL3	LBX2	CYP2U1	HLA-DRB1
STAP2	RPS3A	MRPL12	FITM2	COL6A2	TRAF3IP3

DUSP1	MPZL2	SLC30A1	PRSS23	TYROBP	ALOX5AP
FLT3LG	RPS5	KLRF1	RASSF4	PSTPIP2	IFI27L2
NUDT6	ACSS2	CADM1	OASL	TRDV1	UCP2
VIPR1	CTSH	OASL	GDPD5	SH3BGRL3	ARHGDIB
FXYD5	RPS16	TBC1D2B	TENT5A	ELOVL6	ASB2
ABCA2	RPL35	RPS12	ZNF836	ZNF2	IGFLR1
OBSCN	MPZL1	H2AFY2	CD8B2	KLRC3	SIRPG
CTSL	SAMD1	TBCD	GPD1L	ZNF629	HLA-DPB1
MAP3K1	RPL9	C12orf42	MINCR	TMSB4X	MYO1G
CLHC1	CA2	FITM2	TRGV2	SH3D19	ARPC1B
RPL3	AIG1	MLLT3	ASCL2	MPZL2	MT2A
GIMAP4	ENPP5	GEN1	TSPAN2	H1F0	BLOC1S1
TPT1	SND1-IT1	BBS10	COLGALT2	CKLF	ITGAL
SND1-IT1	IFIT3	ZNF607	GK5	TM6SF1	MAP4K1
P2RY8	RPS8	CPNE8	CIPC	GALNT2	LRRN3
GIMAP7	RPL13	GIPR	SRD5A1	NEURL4	SH3BGRL3
NOS3	B3GALT2	RPS15A	PLEKHO2	LILRB1	CD63
RPS3A	RPL10	KLRG1	MTSS1	ID3	SNAP47
AQP3	NRSN2	PARL	TRGC1	FCGBP	CD3G
FCMR	RPS2	RAB3A	KLRG1	RLN1	DGKZ
HYAL2	INO80B	HHLA3	HLA-B	MTURN	ARL6IP5
RPL13	PRRG2	HLA-B	FAM53B	CD99	PFN1
GIMAP1	KIF9-AS1	GIMAP7	ZNF319	KBTBD6	SLC1A4
RPS8	LMTK3	RPL13	EFCAB5	ABCB1	HLA-A
CCDC191	RPL37A	CCR5	SH3BP5	ARL2	JAML

AMER1	NR2F6	KLF2	NFIA	SMIM3	S100A4
RPS18	TSPAN3	GPR19	PAQR4	USP49	PSMB8
EEF1A1	TPT1	SIPA1	THBS3	MPST	TSPO
SNED1	RPSA	HLA-A	COA7	SPTY2D1OS	HMOX1
KLF3	CYP2U1	KIAA2013	MTERF2	ZCCHC18	SLC9A3R1
RPL39	MIF	HYLS1	HLA-A	LYAR	NELL2
SESN3	EEF1A1	PPM1N	PLEKHA8	OSGEPL1	ANXA5
CST3	CERNA1	SAMD3	FAM78A	CNN3	EVL
RPLP0	RTL10	N4BP3	DECR2	RPL10	ADGRG1
RPL34	RPL39	PTPRM	PHF12	NINL	TMSB4X
FAM210B	RPS19	FAM120C	ZNF564	ZBTB16	SARDH
GVQW3	RPS21	ENPP5	MTBP	DNAJB4	MKI67
VEGFB	EMP3	CHI3L2	RPS4X	CXorf57	FTL
HMGN5	HAGHL	ITGA5	C16orf54	RPS3A	CD2
FXYD7	GPR55	ITM2C	C2orf74	SPATA6L	ENTPD1
FAM161A	ABHD15	SPATA7	SCARB2	POMK	HMGB2
WNT10A	KLHL26	FAU	TRAM2	TPT1	MCM5
PLPP2	ARL2	RPL34	S1PR5	ZNF555	SIT1
C8orf58	MROH8	ZDHHC8	ERMP1	P2RY11	TOX
SLC40A1	EEF1B2	PINLYP	SAMD3	ZNF319	GALNT2
RPS14	RPL34	MDK	ZKSCAN4	ZNF70	KLRB1
POLH	HIST1H2BC	RPS18	SLC9A1	RECQL5	SLC2A8
TNFRSF10D	SMKR1	ZNF365	CD8A	VPS33A	RAC1
THEM4	SARAF	RPL13A	ZBP1	CAPN12	ACP5
RPL9	RABAC1	BTBD9	PDLIM1	TMEM250	ANXA11

LDHB	BEX2	CST3	RPS27	TXNIP	REEP5
FITM2	PERP	FGFBP2	ZNF573	NBPF1	GPR68
C1orf61	PFN2	RABAC1	GIMAP1	SLC35A1	TP73
PI4K2A	FAM213A	SLC25A23	CD8B	RHOC	PSMB9
RPS12	CDK2AP2	ELOVL4	FANCM	DBN1	ANXA6
RPS5	PEBP1	SUSD3	CMTR1	CLNK	RCSD1
ZNF629	GLCE	RPS21	APOBEC3G	PGM3	LIMD2
RPS16	PLCD3	NSG1	HACD4	APOBR	MYL12A
RPS4X	TRIM46	RDM1	ZNF844	SMKR1	ZYX
RPL28	C8orf88	CRYBG2	FBXL14	KIR2DL4	RTP5
RPL35	PDCD4	PTK2B	RPS14	ZBTB42	CCL5
RPL10	SPART	USP31	HLA-C	DMKN	MYO7A
S100A6	RPS18	RPS4X	PAFAH2	C3orf33	CYTH4
TNFSF11	CYB561	TNRC6C	OSBPL7	ATP23	NPDC1
RPS21	CCDC102B	HSD11B1L	PTK2B	CD63	PTPRCAP
RCAN3	MFHAS1	WNT10A	CD68	ATP6V0E2	SYNGR2
CCND3	EXD2	CXCR5	BRICD5	LATS2	TRAPPC1
RPS2	MRPS6	TCP11L2	HAUS3	TGFBR3L	VAMP5
SPATA7	FBXL22	RPL41	TRDC	ZNF888	NLRC3
SPINT2	EGLN2	PGAM5	RPL13A	HDGFL3	RPS24
CRTAP	ZNF510	GIMAP4	MAP4K3	RPS8	CD27
RPL10A	IL15	TRANK1	TMSB4X	IL10RA	CAMK1
ZNF658	SPON1	RPS24	APMAP	FAM89B	ETV7
RPS15A	SSR4	RPS8	VANGL1	RPS5	ACAP1
RPS27	DCLRE1A	TONSL	AGAP3	PMCH	DYNLL1

RPL13A	RHOD	SYNM	ARHGAP25	C8orf88	BST2
RPS9	IFI30	CDKL3	HCG25	KLRC1	TSPAN14
S100A11	LYPD3	CETN3	PANK4	RPS19	ITGB7
RIPOR2	TSPAN4	GLIPR2	IFT172	SLC27A1	SETBP1
RPSA	RPL28	HDAC11	TUBB4A	ARAP3	LAYN
SORL1	PLAUR	PRR5L		P3H4	PYCARD
TLR1	GLO1	CMC1			YPEL1
RAB3D	C17orf67	FAM213A			JAKMIP1
VANGL1	PLCB3	MICB			HCST
C1orf162	RPL7	S1PR1			RPS26
NPIPB2	AHRR				RAC2
B3GAT2	IGFBP3				CYBA
LSR	ZNF449				MPST
RPL41	DOCK7				TRG-AS1
GPR157	HSD11B1L				PECAM1
FLVCR1	RPS7				C1orf53
ICAM2	SNHG7				OAS1
NRROS	CCR6				ARHGAP19
EEF1B2	F2RL1				HLA-DMA
ZMYND19	ZNF626				TMEM140
RPLP1					GSEC
ALDH5A1					LGALS9
TCTN2					C17orf100
PABPC1					RBPJ
CAMK4					NDUFB10

C16orf54					TPX2
NOL4L					KIF11
AIG1					NAP1L4
SARAF					KCNAB2
TMEM173					VCAM1
NAA16					GZMH
LDLRAP1					ZMIZ1
EXD2					RSAD2
RPS19					CCR1
RPL38					SH3BP1
ADD3					FGL2
FAAH2					CCM2
ZBED6CL					U2AF1L4
RBSN					HMGN3
ZNF439					CKAP2
FBXL16					IL2RB
DMKN					PSD4
GINS4					CDCA7
USF2					MT1E
SGSH					PPM1M
					AFAP1L2
					DRAP1
					LMCD1
					CD52
					C12orf75

					PAXX
					WWTR1
					ABI3
					GPAT3
					HLA-C
					CSK
					SEPHS2
					ADGRG5
					ARL3
					RNF213
					PPP1R18
					CAPN1
					TOGARAM2
					HLA-DOA
					FBXW5
					LAT
					FUT8
					PPDPF
					RARRES3
					VNN2
					ITGAE
					BORCS6
					RALGDS
					MT1X
					IL9R

					UBE2L6
					ZNF169
					BCAS4
					ANKRD35
					GPA33
					GYG1
					BIN1
					SMC4
					SPTAN1
					SFXN5
					DHCR24
					HAVCR2
					LRRC28
					TRPS1
					TBC1D10C
					ANP32A
					DNPH1
					TRGV9
					BBC3

Supplemental Table 10. IHC patient characteristics

Panel	Spec num	Date of surgery	Age	Sex	Stage
CD57+ CD8+ T cells	A017	12/16/15	88	F	2
	A075	5/2/14	69	M	2
CD103+ CD8+ T cels	A034	5/22/14	69	M	3
	A056	7/26/13	41	F	3
CD38+ FOXP3+ CD4+ T cells (pTregs)	A060	1/5/11	52	M	3
	A076	3/19/15	78	M	2
CD38- FOXP3+ CD4+ T cells	A004	9/14/11	46	M	2
	A032	7/18/13	68	F	3
